# Modulation of *miR-210* alters phasing of circadian locomotor activity and impairs projections of PDF clock neurons in *Drosophila melanogaster*

**DOI:** 10.1371/journal.pgen.1007500

**Published:** 2018-07-16

**Authors:** Paola Cusumano, Alberto Biscontin, Federica Sandrelli, Gabriella M. Mazzotta, Claudia Tregnago, Cristiano De Pittà, Rodolfo Costa

**Affiliations:** 1 Department of Biology, University of Padova, Padova, Italy; 2 Department of Women and Children’s Health, University of Padova, Padova, Italy; Washington University in Saint Louis School of Medicine, UNITED STATES

## Abstract

Single microRNAs are usually associated with hundreds of putative target genes that can influence multiple phenotypic traits in Drosophila, ranging from development to behaviour. We investigated the function of Drosophila *miR-210* in circadian behaviour by misexpressing it within circadian clock cells. Manipulation of *miR-210* expression levels in the PDF (pigment dispersing factor) positive neurons affected the phase of locomotor activity, under both light-dark conditions and constant darkness. PER cyclical expression was not affected in clock neurons, however, when *miR-210* was up-regulated, a dramatic alteration in the morphology of PDF ventral lateral neuron (LNv) arborisations was observed. The effect of *miR-210* in shaping neuronal projections was confirmed *in vitro*, using a *Drosophila* neuronal cell line. A transcriptomic analysis revealed that *miR-210* overexpression affects the expression of several genes belonging to pathways related to circadian processes, neuronal development, GTPases signal transduction and photoreception. Collectively, these data reveal the role of *miR-210* in modulating circadian outputs in flies and guiding/remodelling PDF positive LNv arborisations and indicate that *miR-210* may have pleiotropic effects on the clock, light perception and neuronal development.

## Author summary

In recent years, the role of microRNAs in regulating the endogenous circadian clock and its rhythmic outputs for behaviour/physiology has been recognized. We have observed that depletion or over-expression of *miR-210* in *Drosophila melanogaster* modulates the phase of locomotor activity, without affecting the molecular oscillation of the pacemaker neurons. Moreover, *miR-210* over-expression dramatically alters the pattern of projections from the PDF-positive Lateral Neurons (LNvs). Differentially expressed genes detected in *miR-210* over-expressing flies implicated circadian processes, neuronal development, and photoreception. Taken together, our findings indicate the involvement of *miR-210* in modulating circadian output and remodelling the projections of PDF clock neurons, and suggest that *miR-210* may have pleiotropic effects on clock, light perception and neuronal development.

## Introduction

Circadian oscillators consist of input pathways that receive external signals, such as light, temperature and food, a central pacemaker that generates rhythmicity, and output pathways that activate downstream rhythmic processes [1–3]. These internal timers allow organisms to adjust their physiology and behaviour to the most appropriate phases of the environmental 24 hour cycle imposed by the Earth’s rotation.

The *Drosophila* core circadian clock is a network of approximately 70 clock neurons per hemisphere, grouped into three major clusters: Dorsal Neurons (DN1, DN2 and DN3), ventral Lateral Neurons (small (s) and large (l) LNvs) and dorsal Lateral Neurons (LNds). Four out of five s-LNvs and the l-LNvs express Pigment Dispersing Factor (PDF), a neuropeptide which is involved in shaping locomotor activity in free-running conditions under constant darkness (DD). Under light-dark (LD) conditions PDF sets the phase of evening activity and regulates sleep [4–6]. The PDF-positive LNvs send their projections to different parts of the brain, the s-LNvs reaching the dorsal area, while the l-LNvs send their projections to the contralateral part of the brain and arborise in the Optic Lobe (OP), a structure that processes visual information from retinal or extraretinal photoreceptors [7,8]. Under LD 12:12, flies display two bouts of locomotor activity, one in the morning, starting before Lights-ON, and the other in the evening, before Lights-OFF. Under DD conditions, flies remain rhythmic with a period of about 24h. Over the past fifteen years, several research groups have demonstrated how different neuronal clusters are responsible for specific behavioural activity, under LD, DD or LL (constant light). In a simplified model, the s-LNvs are capable of driving dawn activity and sustaining rhythmicity under DD whereas a subset of LNds and the 5^th^ s-LNv are responsible for dusk activity and for sustaining rhythmicity under LL [9–11].

At the molecular level, interlocked feedback loops generate the rhythmic expression of clock genes in the three neuronal clusters mentioned above. The circadian transcription factors CLOCK (CLK) and CYCLE (CYC) form a heterodimer (CLK-CYC) that activates the expression of the clock genes *period* (*per*) and *timeless* (*tim*). Following complex phosphorylation and degradation dynamics, PER and TIM eventually accumulate, enter the nucleus, and repress the transcriptional activity of the CLK-CYC heterodimer, thus inhibiting their own transcription. CLK-CYC also drive the expression of *vrille* (*vri*), *Par Domain Protein 1* ε (*pdp1*ε) and *clockwork orange* (*cwo*). VRI and PDP1ε are part of a second intersecting regulatory loop which controls *Clk* gene transcription and reinforces the circadian molecular oscillation, while CWO acts as a repressor of CLK-CYC-mediated transcription. Post-translational mechanisms, driven by kinases such as DOUBLETIME (DBT), SHAGGY (SGG), CASEIN KINASE2 (CK2), ubiquitin-ligases such as SUPERNUMERARY LIMBS and CULLIN-3, and phosphatases such as PROTEIN PHOSPHATASE 1 and 2A, play important roles in regulating PER, TIM and CLK stability [12]. The result is the cyclical expression of several clock genes and hundreds of clock-controlled genes (CCGs), which generate rhythmic physiological and behavioural outputs. Post-transcriptional post-translational regulation is therefore crucial for circadian clock functioning [13–17].

MicroRNAs (miRNAs) are a class of small ~22 nucleotide non-coding RNAs that act as important post-transcriptional regulators [18,19]. They negatively control gene expression by targeting mRNAs, mostly at the 3’ untranslated region (3’-UTR) and triggering either translational repression or RNA degradation [20,21]. It has been estimated that approximately 30% of genes are regulated by at least one miRNA [22,23] so miRNAs are implicated in a wide variety of biological processes, including differentiation [24], apoptosis [25], lipid metabolism [26], viral infections [27], tumorigenesis [28–30] and neurodegeneration [31,32] and not surprisingly, complete deficiency of miRNAs is incompatible with life [33,34].

Several studies have demonstrated that miRNAs are involved in post-transcriptional regulation of fly clock or clock-controlled genes [35,36]. The over-expression of *bantam* miRNA in clock cells was shown to induce period lengthening by regulating the *Clk* 3’UTR [37]. *miR-279* influences the behavioural output of flies by directly modulating the *Unpaired* gene, which is involved in the JACK/STAT pathway [38]. *cwo* is modulated by *let-7* miRNA which, in turn, is regulated by the clock via the prothoracicotropic hormone signalling pathway, which stimulates the production of the molting hormone ecdysone [39]. Furthermore, it has been recently demonstrated that *miR-124* is involved in the regulation of the phase of locomotor activity [40,41] and *miR-276a* regulates molecular and behavioural rhythms by inhibiting the expression of the clock gene *timeless* [42]. In spite of the role of several miRNAs in circadian phenotypes, most miRNAs from fly heads show little or no circadian oscillations [43]. Yet one of these ‘non-cycling’ miRNAs, *miR-210*, which is up-regulated in *cyc*^*01*^ flies, does indeed show cyclical expression levels within the PDF pacemaker neurons under LD conditions, with a peak of expression at *Zeitgeber* Time 6 (ZT6) [44].

Our study focuses on *miR-210* and reveals how misexpression causes pleiotropic effects on circadian activity phase, on shaping projections of PDF-expressing LNvs, and on motion detection.

## Results

### *miR-210* modulates the circadian phase activity of flies

We analysed *miR-210* knock-out mutants (*yw(Ti-Gal4)miR-210*^*KO*^) and flies overexpressing the extended region (153 nt) of the *pre-miR-210* (*UAS-miR-210*) in clock cells using the *tim-gal4(UAS)* driver (hereafter termed *tim-Gl4(U)*). Over-expression of mature miRNA was evaluated by qRT-PCR under LD12:12 in adult fly brains dissected at ZT0 and on the 3rd day of DD at CT72 and was ~15 fold greater than wild-type (S1A Fig). No *miR-210* expression was detected in the *yw(Ti-Gal4)miR-210*^*KO*^ strain (S1B Fig).

**Fig 1:**
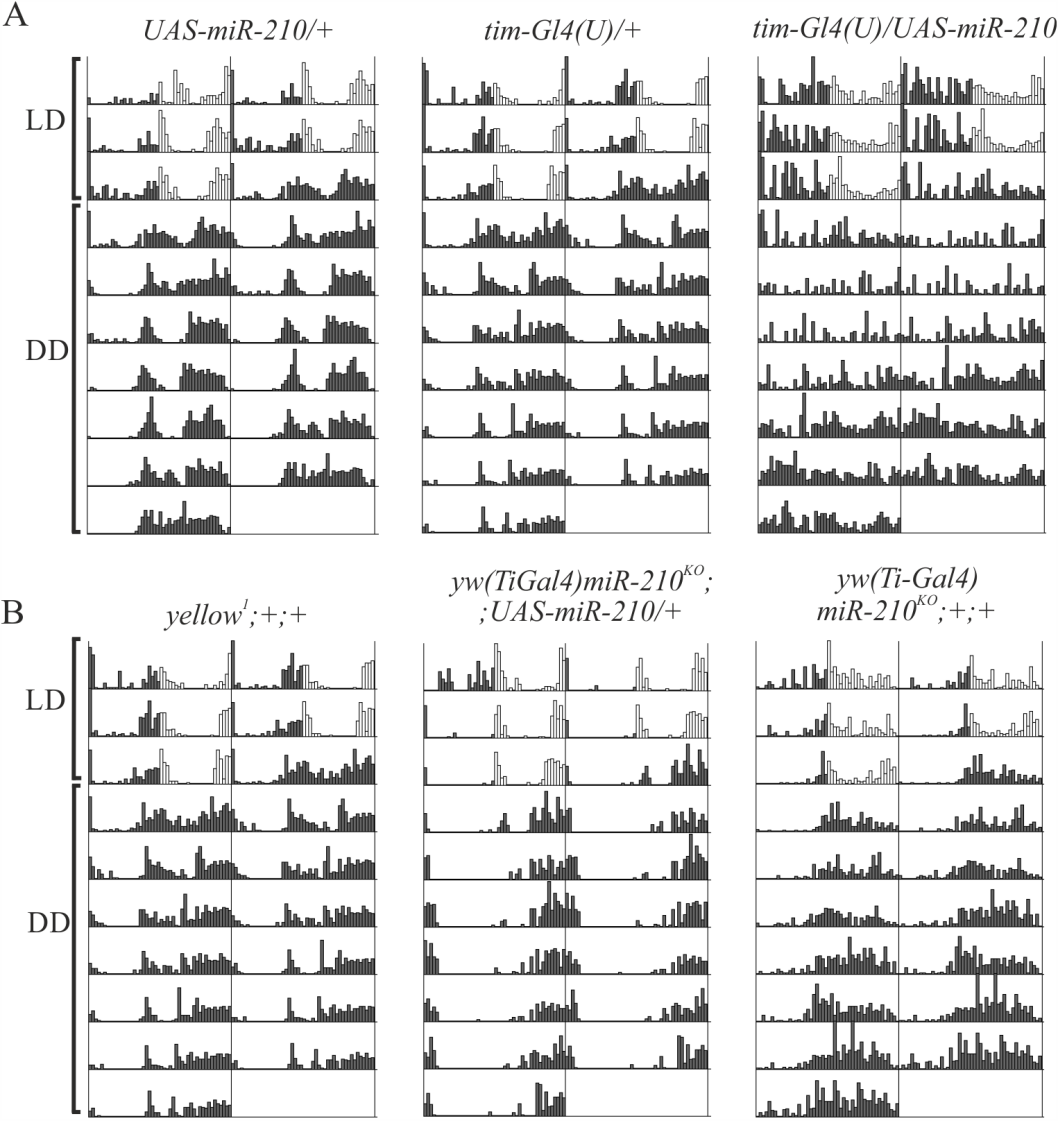
Locomotor activity of flies with impaired miR-210 expression. Double-plotted locomotor activity of a single representative fly per genotype, recorded for 3 complete days under LD and 7 complete days under DD. (A) Most flies that over-expressed *miR-210* in clock cells (*tim-Gl4(U)/ /UAS-miR-210*) became arrhythmic under DD, compared to controls (*UAS-miR-210/+* and *tim-Gl4(U)/+*). (B) *yw(Ti-Gal4)miR-210*^*KO*^*;+;+* flies were rhythmic under DD as were *yellow*^*1*^*;+;+* and *yw(Ti-Gal4)miR-210*^*KO*^*;UAS-miR-210/+* rescued flies, here considered as controls. (dark grey: dark phase; white: light phase).

Under DD, over-expression of *miR-210* (*tim-Gl4(U)/UAS-miR-210*) led to disruption of locomotor activity cycles with 70% arrhythmicity. The remaining rhythmic individuals showed a period which was lengthened by approximately 1 h but with 5 h phase delay (Fig 1A, Tables 1 and 2). Knockout of *miR-210* did not alter rhythmicity of flies (Fig 1B, Table 1) but significantly advanced circadian phase by ~6 h (Table 2).

**Table 1:** Locomotor activity of flies with *miR-210* impaired expression in LD and DD.

Genotype	N° tot	N° alive	N°R	% R	τ		SEM	% MA	% EA
*w;tim-Gl4(U)/UAS-miR-210*	137	108	33	**30.56**	24.85	±	0.10	**41.67**	75.00
*w;UAS-miR-210/+*	138	128	117	91.41	23.84	±	0.04	94.53	98.44
*w;tim-Gl4(U)/+*	187	162	135	83.3	23.66	±	0.05	87.04	98.77
*w*^*1118*^	108	95	89	93.68	24.04	±	0.07	96.84	98.95
*yw(Ti-Gal4)miR-210*^*KO*^*;+;+*	38	37	33	89.19	24.09	±	0.10	100	83.78
*yellow*^*1*^*;+;+*	63	63	62	98.41	24.07	±	0.06	61.90	98.41
*yw(Ti-Gal4)miR-210*^*KO*^*;UAS-miR-210/+*	109	106	101	95.28	24.03	±	0.05	73.58	94.34
*yw(Ti-Gal4)miR-210*^*KO*^*;UAS-miR-210/+; cry-Gal80/+*	60	59	34	**57.63**	23.36	±	0.06	59.32	88.14
*yw(Ti-Gal4)miR-210*^*KO*^*;UAS-miR-210/pdf-Gal80*	26	24	20	83.33	23.76	±	0.12	95.83	100
*yw;;cry-Gal80/+*	23	23	21	91.30	24.09	±	0.09	69.57	95.65
*yw;pdf-Gal80/+*	16	15	15	100	24.26	±	0.07	86.67	93.33

Male flies were monitored for three days in LD 12:12 and seven days in DD. *w;tim-Gl4(U)/+* and *w;UAS-miR-210/+* flies are parental controls for the *miR-210* over-expressing flies (*w;tim-Gl4(U)/UAS-miR-*210), while *yellow*^*1*^*;+;+*, and *yw;;cry-Gal80/+* plus *yw;pdf-Gal80/+* flies are controls for *miR-210* KO (*yw(Ti-Gal4)miR-210*^*KO*^*;+;+*) and *miR-210* KO-rescued flies (*yw(Ti-Gal4)miR-210*^*KO*^*;UAS-miR-210/+*, *yw(Ti-Gal4)miR-210*^*KO*^*;UAS-miR-210/+; cry-Gal80/+* and *yw(Ti-Gal4)miR-210*^*KO*^*;UAS-miR-210/pdf-Gal80*) respectively. N: number of tested flies per genotype. R: rhythmic flies; MA: morning anticipation; EA: evening anticipation. Period values (τ) were averaged over all rhythmic flies per genotype. MA and EA were detected, fly-by-fly, by examining the bout of activity prior to light transitions as in [45]. The experiments were performed at 23°C.

**Table 2:** Locomotor activity phase in DD.

Genotype	DD ϕ (N)		SEM
*w;tim-Gl4(U)/UAS-miR-210*	**14.82** (11) ^a^	±	0.22
*w;UAS-miR-210/+*	8.63 (49)	±	0.46
*w;tim-Gl4(U)/+*	9.31 (34)	±	0.40
*yw (Ti-Gal4)miR-210*^*KO*^*;+;+*	**1.65** (33) ^b^	±	0.50
*yellow*^*1*^*;+;+*	8.73 (62)	±	0.46
*yw (Ti-Gal4)miR-210*^*KO*^*;UAS-miR-210/+*	7.98 (81) ^c^	±	0.32
*yw (Ti-Gal4)miR-210*^*KO*^*;UAS-miR-210/+; cry-Gal80/+*	**3.83 (33)** ^d^	±	0.52
*yw(Ti-Gal4)miR-210*^*KO*^*;UAS-miR-210/pdf-Gal80*	**5.10 (20)** ^e^	±	0.98
*yw;;cry-Gal80/+*	10.10 (21)	±	0.56
*yw;pdf-Gal80/+*	12.08 (13)	±	0.95

^a^ p<0.005 *vs* parental controls (*w;UAS-miR-210/+* and *w;tim-Gl4(U)/+*)

^b^ p<0.005 *vs yellow*^*1*^*;+;+*

^c^ p<0.005 *vs yw (Ti-Gal4)miR-210*^*KO*^*;+;+*, and ns *vs UAS-miR-210/+*

^d^ p<0.005 *vs yw (Ti-Gal4)miR-210*^*KO*^*;UAS-miR-210/+* and *yw;;cry-Gal80/+*

^e^ p<0.005 vs *yw (Ti-Gal4)miR-210*^*KO*^*;UAS-miR-210/+* and *yw;pdf-Gal80/+*. Mann-Whitney U Test was performed.

**Fig 2:**
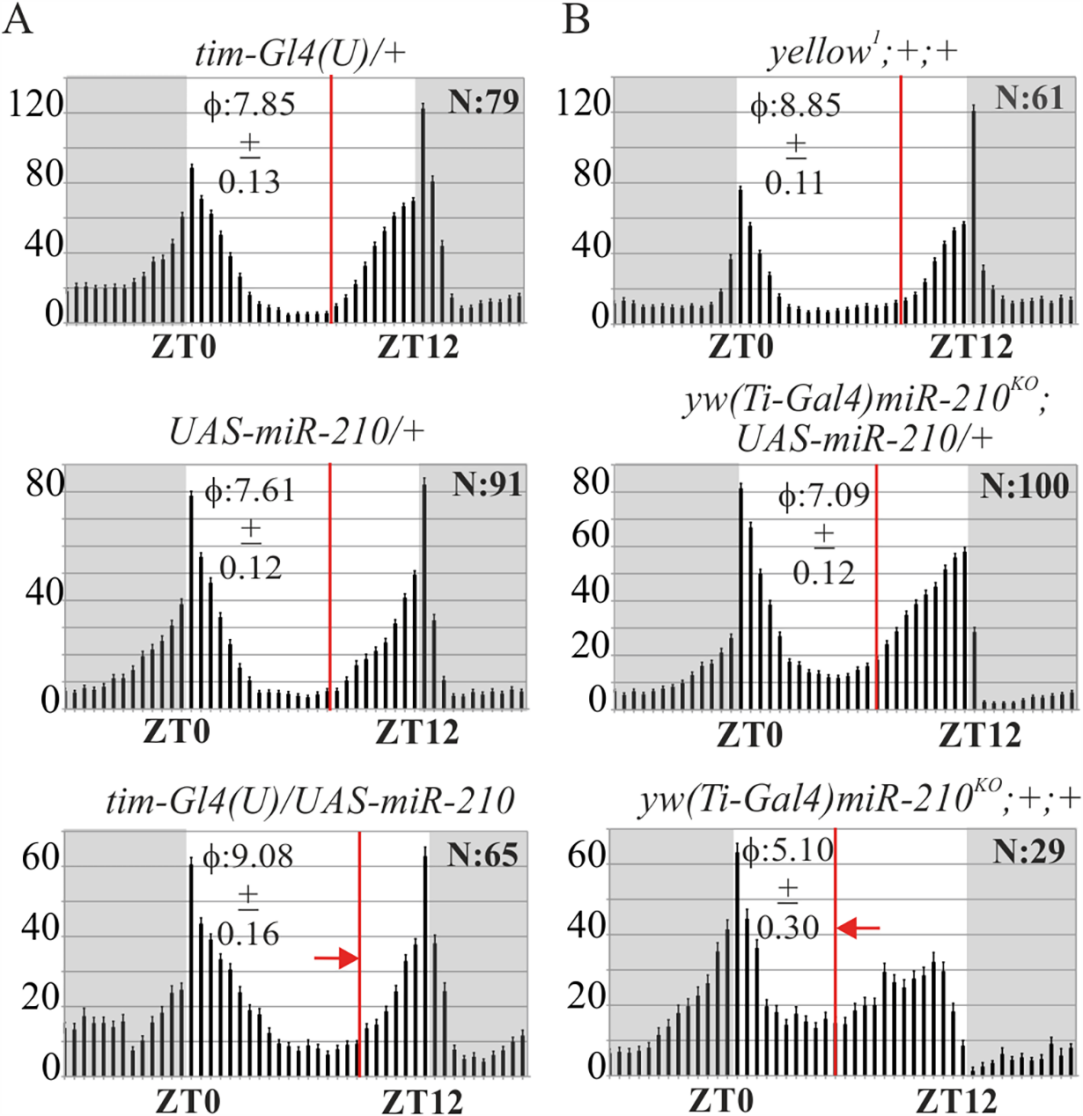
Locomotor activity of flies with *miR-210* altered expression in LD (average activity of three full days displayed over the 24 hours). (A) *miR-210* over-expression in clock cells (*tim-Gl4(U)/UAS-miR-210*) delayed the evening activity phase onset (red arrow), compared to controls (*tim-Gl4(U/+* and *UAS-miR-210*/+). (B) *miR-210* KO flies (*yw(Ti-Gal4)miR-210*^*KO*^*;+;+*) displayed a robust morning anticipation and an advanced evening activity (red arrow) compared to controls (*yellow*^*1*^*;+;+* and *yw(Ti-Gal4)miR-210*^*KO*^*; UAS-miR-210/+*). *miR-210* rescued flies showed a wild-type evening activity phase. ϕ: evening phase activity onset ± SEM (red lines); N: number of flies; y axis: locomotor activity (beam crosses/30 min) ± SEM; grey boxes: dark phase.

Under LD12:12, flies over-expressing *miR-210* in clock cells lost their ability to anticipate the lights-ON transition (as shown by Morning Index (MI) and Morning Anticipation (MA) values) (Tables 1 and 3) and delayed the evening activity onset (Fig 2A, Table 3). By contrast, *yw(Ti-Gal4)miR-210*^*KO*^ flies showed an advanced evening activity onset, while morning anticipation was not affected (Fig 2B, Tables 1–3). These results suggest that *miR-210* levels can influence the rhythmicity and the circadian phase of activity under both LD and DD conditions.

**Table 3:** Morning index and evening phase onset in LD.

Genotype	MI (N)		SEM	E Onset (N)		SEM
*w;tim-Gl4(U)/UAS-miR-210*	**0.019** (108) ^a^	±	0.01	**9.08** (65) ^b^	±	0.16
*w;UAS-miR-210/+*	0.235 (128)	±	0.01	7.61 (91)	±	0.12
*w;tim-Gl4(U)/+*	0.106 (162)	±	0.01	7.85 (79)	±	0.13
*yw (Ti-Gal4)miR-210*^*KO*^*;+;+*	0.243 (37)	±	0.02	**5.07** (29) ^c^	±	0.30
*yellow*^*1*^*;+;+*	0.029 (63)	±	0.02	8.85 (61)	±	0.11
*yw (Ti-Gal4)miR-210*^*KO*^*;UAS-miR-210/+*	0.183 (109)	±	0.01	**7.09** (100) ^d^	±	0.12
*yw (Ti-Gal4)miR-210*^*KO*^*;UAS-miR-210/+; cry-Gal80/+*	0.046 (59) ^e^	±	0.02	7.13 (53) ^f^	±	0.18
*yw(Ti-Gal4)miR-210*^*KO*^*;UAS-miR-210/pdf-Gal80*	0.247 (24)	±	0.02	**6.42** (24) ^g^	±	0.20
*yw;;cry-Gal80/+*	0.080 (23)	±	0.03	8.85 (22)	±	0.27
*yw;pdf-Gal80/+*	0.195 (14)	±	0.04	7.79 (14)	±	0.33

^a,b^ p<0.005 *vs* both parental controls

^c^ p<0.005 ^vs^ *yellow*^*1*^;+;+

^d^ p<0.005 *vs yw(Ti-Gal4)miR-210*^*KO*^*;+;+* and *yellow*^*1*^*;+;+*, and p<0.01 *vs UAS-miR-210/+*

^e^ ns *vs yw;;cry-Gal80/+*

^f^ ns *vs yw (Ti-Gal4)miR-210*^*KO*^*;UAS-miR-210/+* and p<0.005 *vs yw;;cry-Gal80/*

^g^ p<0.005 *vs yw (Ti-Gal4)miR-210*^*KO*^*;UAS-miR-210/+* and *yw;pdf-Gal80/+*. Mann-Whitney U Test was performed. MI: Morning index. N: number of flies analysed. The E Onset (LD evening activity onset, ZT) was calculated as described in the Methods.

The DD ϕ (peak of locomotor activity phase, CT) was calculated as described in the Methods. N: number of tested flies.

### Characterization of *miR-210* expression in the brain

**Fig 3:**
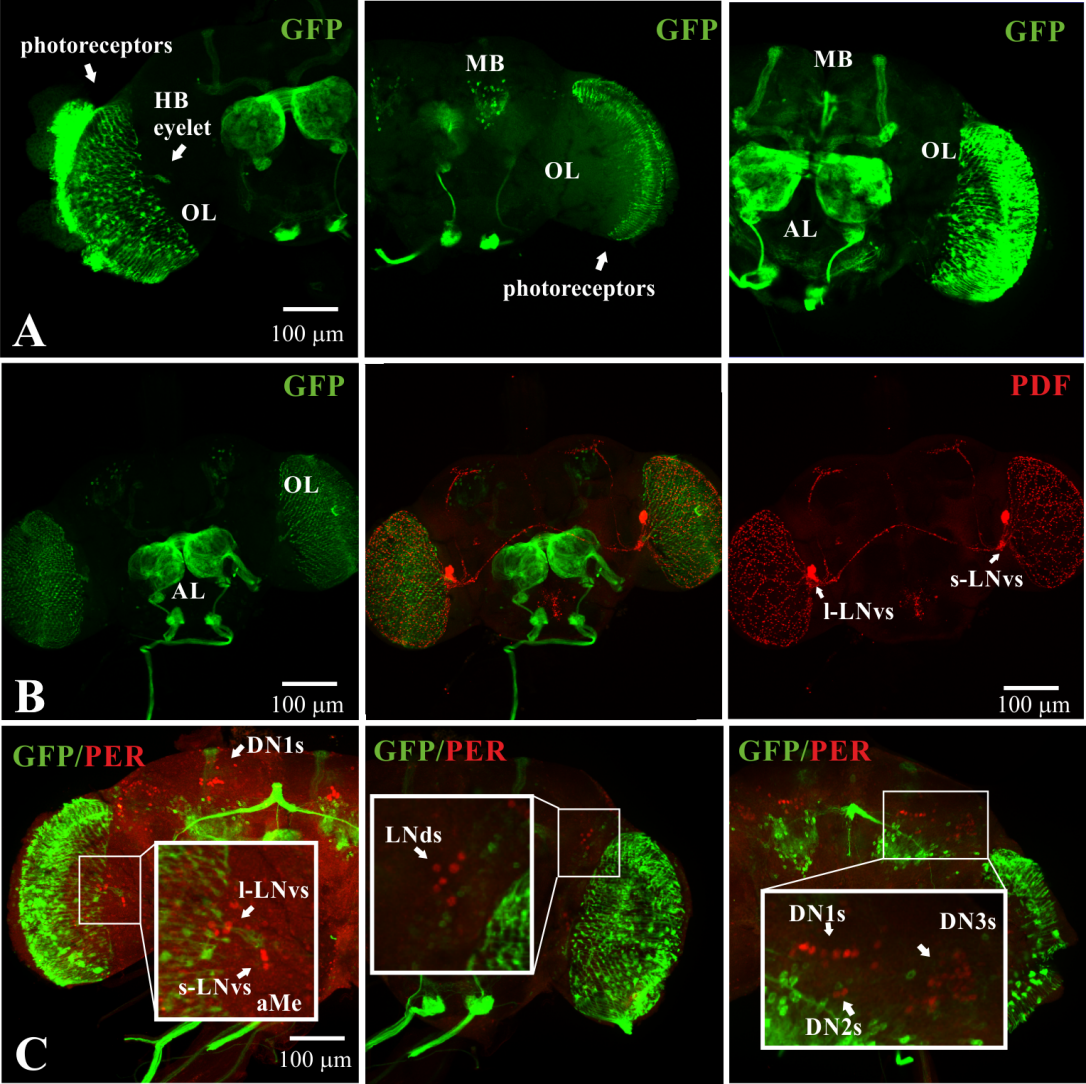
*yw(Ti-Gal4)miR-210*^*KO*^ expression in Drosophila brain. *yw(Ti-Gal4)miR-210*^*KO*^ flies were crossed with the *UAS-cd8-GFP* flies. The progeny was collected in between ZT0 and ZT3. Stack of confocal images of (A) *miR-210-*driven GFP expression in photoreceptors, in the Optic Lobe (OL), Hofbauer-Buchner eyelet (H-B eyelet), Antenna Lobe (AL), and Mushroom Bodies (MB), (B) Double GFP-PDF and (C) GFP-PER staining. (aMe: accessory Medulla). (s-LNvs: small ventral Lateral Neurons; l-LNvs: large ventral Lateral Neurons; 5^th^-LNvs: 5^th^ ventral Lateral Neuron; LNds: dorsal Lateral Neurons; DN1s: Dorsal Neurons 1; DN2s: Dorsal Neurons 2; DN3s: Dorsal Neurons 3).

In the *yw(Ti-Gal4)miR-210*^*KO*^ strain, the sequence of *miR-210* is replaced by the *Gal4* cDNA [46]. These flies were crossed with flies carrying the *UAS-cd8-GFP* transgene, in order to detect the expression pattern of the endogenous *miR-210* promoter. GFP signal was detectable in the optic lobe (OL), Antenna Lobe (AL), photoreceptors, Mushroom Bodies (MB) and in the Hofbauer-Buchner eyelet (HB-eyelet) (Fig 3A). GFP expression was not detected in clock neurons (Fig 3B and 3C), although *miR-210* expression has been previously reported in these cells by qRT-PCR [44]. No differences in the expression pattern were observed between sexes.

### *miR-210* modulates the circadian locomotor activity of flies when expressed in clock neurons

To identify the subset of neurons responsible for the modulation of locomotor activity by *miR-210*, the Gal4 lines *yw(Ti-Gal4)miR-210*^*KO*^, *cry-Gal4*, *Gal1118-Gal4* (which essentially marks the PDF positive cells and, very weakly, some other clock neurons and non-clock cells [47]), *pdf-Gal4* and *C929-Gal4* (which marks l-LNvs as the only clock cells as also some peptidergic non-clock cells [10,48]) were used to set up crosses with *UAS-miR-210* flies. In the progeny, the evening activity onset was delayed in all the genotypes compared to controls, with the exception of *yw(Ti-Gal4)miR-210*^*KO*^ and *C929-Gal4* (in the case of *yw(Ti-Gal4)miR-210*^*KO*^ only female progeny were analysed as the construct maps to the X chromosome) (S2 Fig, S1 Table). The *cry-Gal4* driver was the only line that phenocopied the behavioural arrhythmicity in DD induced by over-expression of *miR-210* with *tim-Gl4(U)* (S2 Table). Broad expression of *miR-210* with *tubulin-Gal4*, *repo-Gal4* (glial cells) and *elav-Gal4* (pan-neuronal driver) resulted in lethality. Expressing *miR-210* in *miR-210* KO males (*yw(Ti-Gal4)miR-210*^*KO*^*;UAS-miR-210*) affected the evening phase in LD and DD, restoring a wild-type phenotype (Figs 1B and 2B, Tables 1–3). Indeed, the advanced evening onset observed in *miR-210* KO flies was delayed. These findings reveal that the expression of *miR-210* in the OL, MB, AL, photoreceptors and HB eyelet, and also in clock neurons, although not in the l-LNvs, was sufficient to delay the phase of the evening onset of flies in LD.

**Fig 4:**
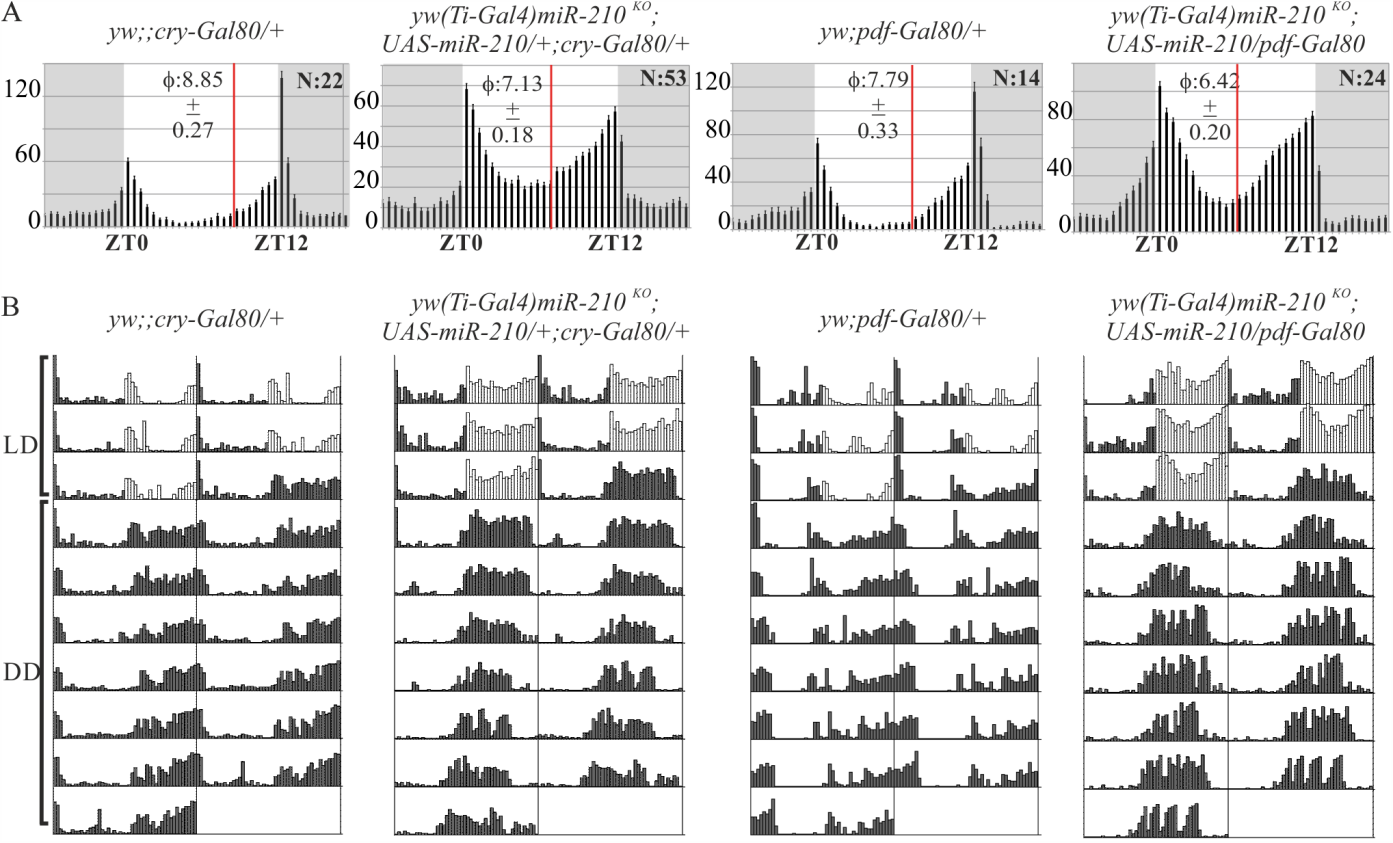
Locomotor activity of flies with *miR-210* depletion in clock neurons. (A) LD locomotor activity profile (average activity of three full days displayed over the 24 hours) of *yw(Ti-Gal4)miR-210*^*KO*^*;UAS-miR-210/+* flies combined with the *cry-Gal80* or the *pdf-Gal80* repressor transgenes, compared to controls (yw;;*cry-Gal80/+* or the yw;*pdf-Gal80* /+). ϕ: evening phase activity onset ± SEM (red lines); N: number of flies; y axis: activity (beam crosses/30 min) ± SEM; grey boxes: dark phase. (B) Double-plotted locomotor activity of a single representative fly per genotype, recorded for 3 complete days under LD and 7 complete days under DD. Flies with no *miR-210* expression in CRY or PDF positive neurons severely phase-advanced their activity under DD.

We depleted *miR-210* expression in CRY positive and PDF positive clock neurons, combining the *yw (Ti-Gal4)miR-210* ^*KO*^ rescued flies with the *cry-Gal80* or *pdf-Gal80* repressors. In these flies, the phase of the evening onset was not affected (*yw (Ti-Gal4)miR-210*^*KO*^*;UAS-miR-210/+; cry-Gal80/+*), or was weakly advanced (*yw(Ti-Gal4)miR-210*^*KO*^*;UAS-miR-210/pdf-Gal80*) compared to controls (Fig 4A, Table 3). On the other hand, under DD conditions flies showed a significantly advanced locomotor activity phase (Fig 4B, Table 2), a phenotype previously observed in *miR-210* KO flies (Table 2). In addition, half of the flies without *miR-210* expression in CRY positive clock neurons (*yw (Ti-Gal4)miR-210*^*KO*^*;UAS-miR-210/+; cry-Gal80/+*) became arrhythmic under DD, similar to the observation in *tim-Gl4(U)* or *cry-Gal4 miR-210* over-expressing flies (Table 1, S2 Table). Altogether, these data confirmed that *miR-210* is indeed expressed in clock neurons, where it exerts a prominent role in setting locomotor phase in the absence of external cues, as well as supporting rhythmicity.

### *miR-210* modulates levels of PER in clock neurons

**Fig 5:**
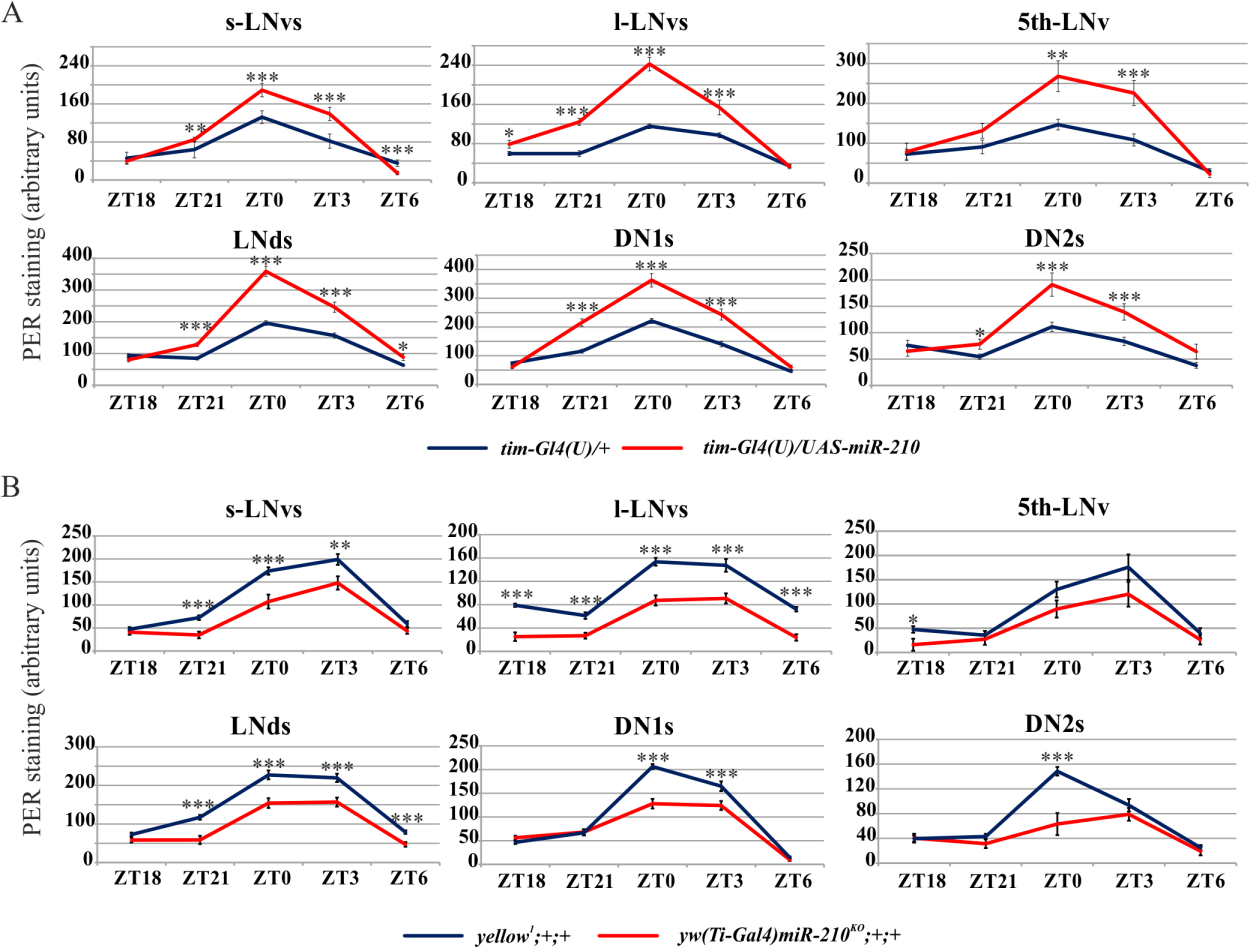
PER expression levels in flies with *miR-210* altered expression in LD. Flies were entrained for 3 days. PER-PDF staining was performed on whole adult male brains dissected at the indicated time points. (A) *tim-Gl4(U)/UAS-miR-210* over-expressing flies and controls (*tim-Gl4(U)/+*). (B) *yw(Ti-Gal4)miR-210*^*KO*^*;+/+* flies and controls (*yellow*^*1*^*;+/+*). (s-LNvs: small ventral Lateral Neurons; l-LNvs: large ventral Lateral Neurons; 5^th^-LNvs: 5^th^ ventral Lateral Neuron; LNds: dorsal Lateral Neurons; DN1s: Dorsal Neurons 1; DN2s: Dorsal Neurons 2). Data for each ZT were compared by t-test: *** p<0.005, ** p<0.01, * p<0.05.

To determine whether the advanced or delayed activity phase reflected molecular changes in clock protein oscillations in clock neurons, we performed PER staining in *miR-210* over-expressing flies and *miR-210* KO flies. Whole adult fly brains were dissected at five different ZT time points, under LD. Although manipulation of *miR-210* levels did not affect PER cycling (Fig 5A and 5B), a higher level of protein was detected in all canonical clock neurons of *tim-Gl4(U) miR-210* over-expressing flies compared to controls (Fig 5A). Conversely, *miR-210* KO led to a reduction in PER levels in clock neurons (Fig 5B).

Since *miR-210* over-expression in clock cells reduced the rhythmicity of flies under DD, we also quantified PER in *tim-Gl4(U)/UAS-miR-210* flies under these conditions. The oscillations of PER persisted in clock neurons in *tim-Gl4(U)/UAS-miR-210* flies (except for the l-LNvs, [49,50]), while the expression levels were similar to those of controls (S3 Fig). The higher level of PER in LD was interpreted as being strictly related to the presence of light since it was missing or attenuated under DD. Taken together, these data indicate that *miR-210* impacts on PER levels in clock neurons, but not on its cycling.

### *miR-210* affects projections and shape of the PDF-expressing neurons

**Fig 6:**
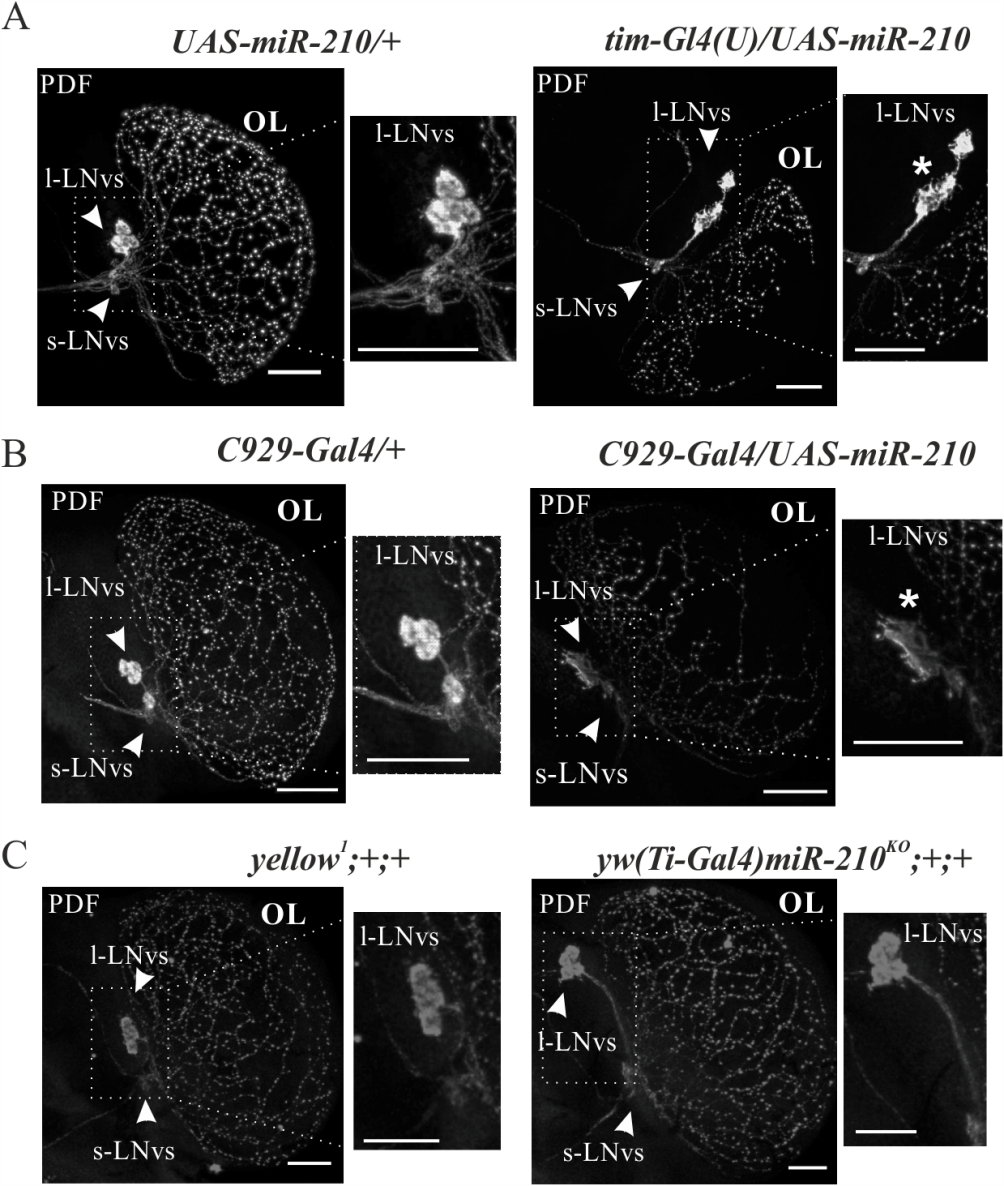
PDF staining of l-LNvs. Whole brains were collected at ZT0 and PDF was detected. Confocal stack images representing l-LNvs arborisations and morphology. (A) Flies over-expressing miR-210 in all clock cells (*tim-Gl4(U)/UAS-miR-210*) showed aberrant PDF arborisations in the optic lobes compared to controls (*UAS-miR-210/+*). Cell bodies of the l-LNvs exhibited a star shaped phenotype (marked with an asterisk). (B) Targeted miR-210 over-expression in l-LNvs clock neurons (*C929-Gal4/UAS-miR-210*) and controls (*C929-Gal4/+*). The l-LNvs appeared to have the same defects in their projections to the optic lobes and in their cell bodies, as in *tim-Gl4(U)/UAS-miR-210* flies (marked with an asterisk). (C) No l-LNvs arborisation defects were detected in *yw(Ti-Gal4)miR-210*^*KO*^*;+/+* flies compared to controls (*yellow*^*1*^*;+/+*). (OL: Optic Lobe; scale bar: 25 μm).

**Fig 7:**
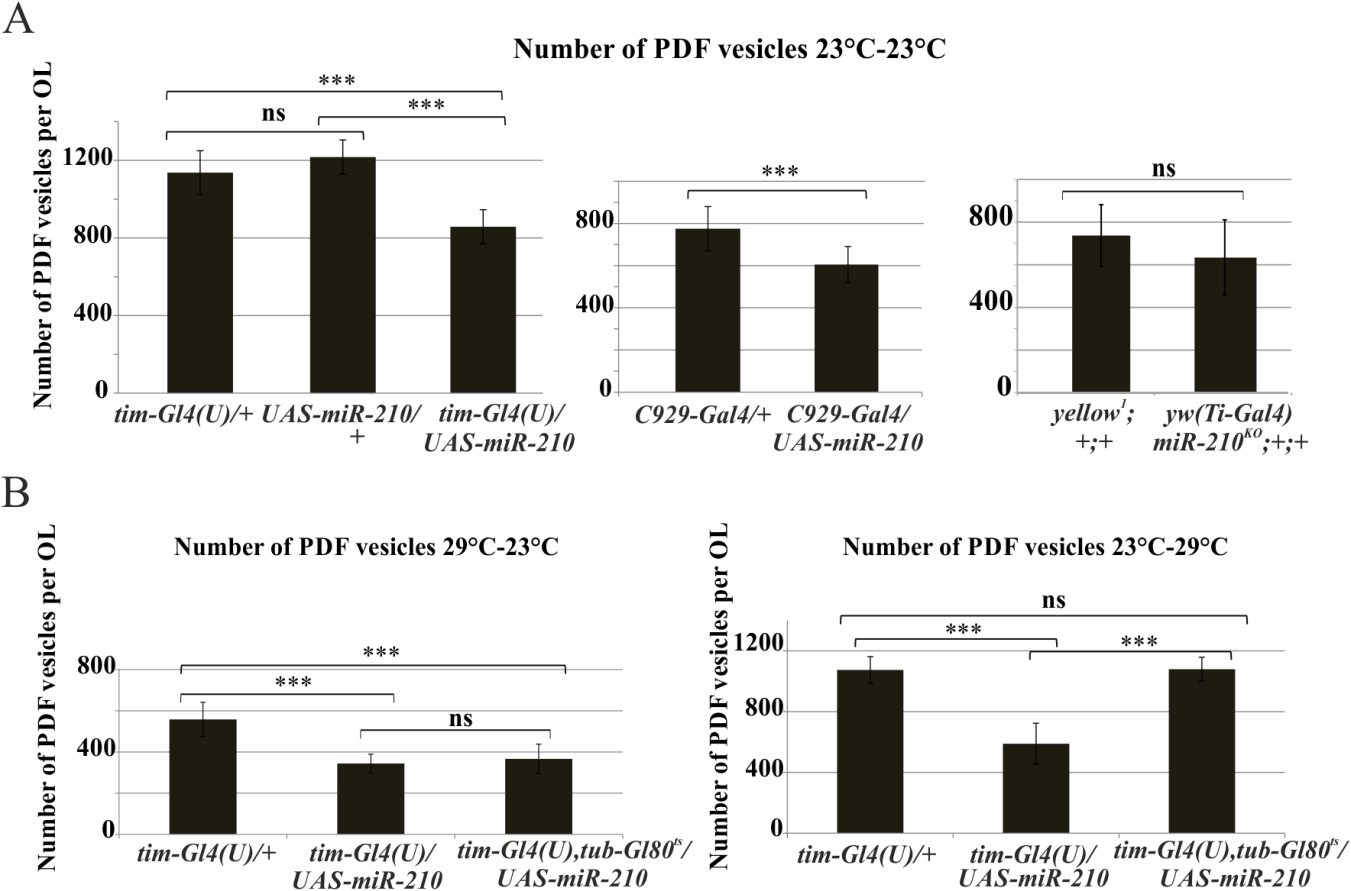
PDF vesicles quantification in the optic lobe. (A) Number of PDF vesicles in (A) miR-210 over-expressing flies (*tim-Gl4(U)/UAS-miR-210* and *C929-Gal4/UAS-miR-210*) and *yw(Ti-Gal4)miR-210* ^*KO*^*;+/+*, compared to their controls *(tim-Gl4(U)/+*, *UAS-miR-210/+*, *C929-Gal4/+* and *yellow*^*1*^*;+/+*), and (B) in flies with miR-210 over-expression (*tim-Gl4(U)*,*tub-Gl80*^*ts*^*/UAS-miR-210/+*) restricted only during development (29°C-23°C), or only during adulthood (23°C-29°C), with respect to positive (constitutive over-expression, *tim-Gl4(U)/UAS-miR-210*) and negative (*tim-Gl4(U)/+*) controls. Flies were dissected at ZT0. Over-expression was modulated by using the Gal4-Gal80^ts^-UAS ternary system. Mean ± SD. t-test: *** p<0.005, ns: not significant.

In parallel with the detection of PER, brains were also stained with PDF antibody. Flies over-expressing *miR-210* (*tim-Gl4(U)/UAS-miR-210*) exhibited aberrant PDF-positive arborisations of the l-LNvs in the optic lobe (OL), as well as an altered star shaped morphology of the cell body (resembling filopodia and lamellipodia protrusions) (Fig 6A). This phenotype was observed when the *tim-Gl4(U)*, the *C929-Gal4* or the *gal1118-Gal4* drivers were employed (Fig 6A and 6B and S4A Fig, respectively). As expected, given the lack of PDF projections in this area, the number of vesicles in the OL was reduced in the flies over-expressing the miRNA, compared to controls (Fig 7A). A double PDF-GFP staining performed in brains over-expressing GFP and *miR-210* under control of *Gal1118-Gal4* (S5 Fig) revealed that *miR-210* is involved in defining the pattern of l-LNv projections in the OL and shaping their cell bodies, rather than affecting the neuropeptide localization (S5 Fig). No differences in l-LNvs arborisations were detected in *yw(Ti-Gal4)miR-210*^*KO*^ and *pdf-Gal4/UAS-miR-210* flies (Fig 6C, S4B and S4C Fig).

**Fig 8:**
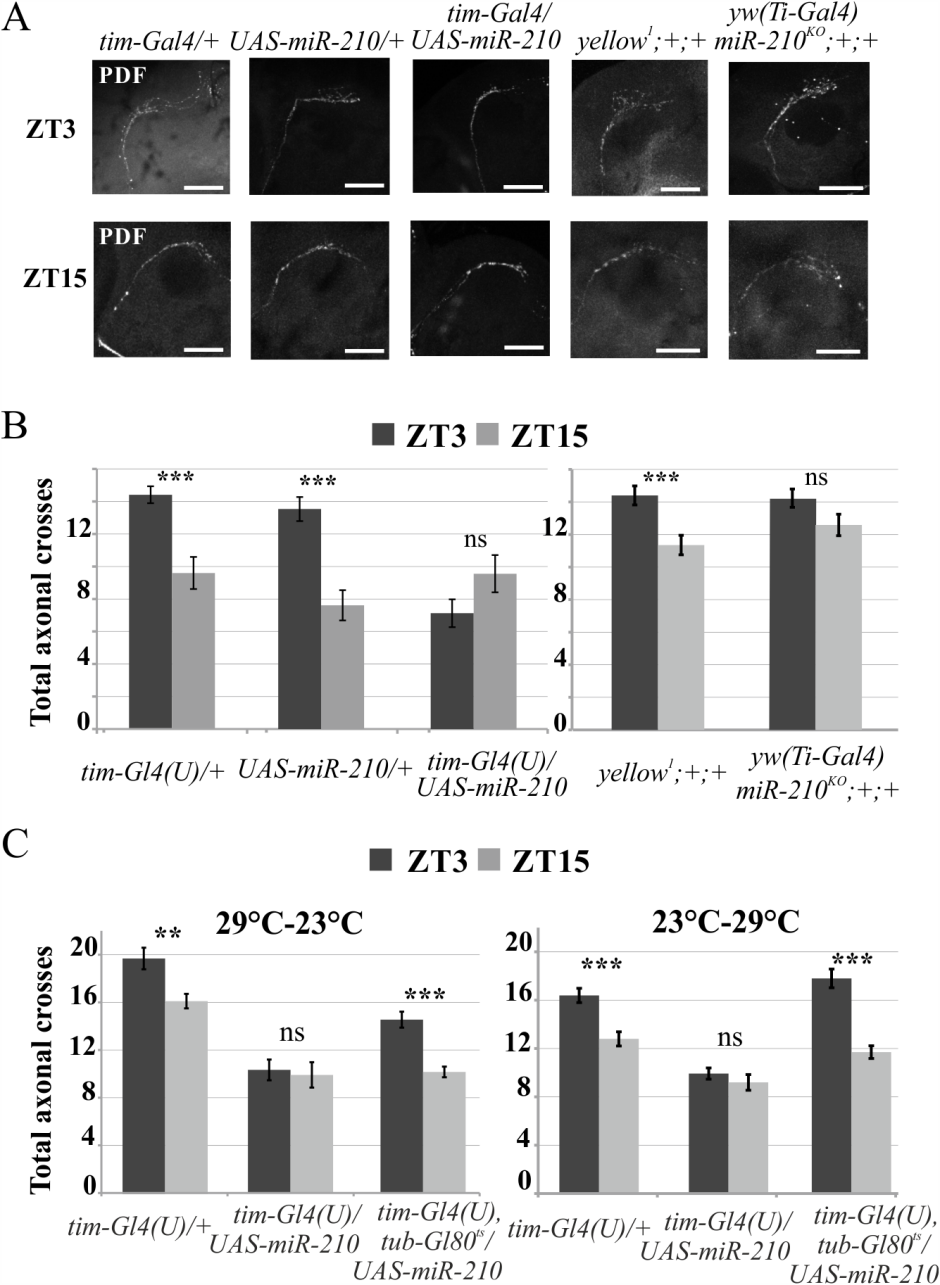
Effects of *miR-210* manipulation on s-LNvs axon termini. (A) Confocal stack images representing the s-LNvs organization at ZT3 (defasciculate state) and ZT15 (fasciculate state). PDF immunostaining was performed on miR-210 over-expressing flies (*tim-Gl4(U)/UAS-miR-210*) or on *yw(Ti-Gal4)miR-210*^*KO*^*;+;+* flies and their controls (*tim-Gl4(U)/+*, *UAS-miR-210/+* and *yellow*^*1*^*;+/+*). Scale: bar 50 μm. (B) Quantification of total s-LNvs axonal crosses of the aforementioned genotypes, performed on confocal stacks images, as described in the Methods. (C) Quantification of total s-LNvs axonal crosses in flies with *miR-210* over-expression (*tim-Gl4(U)*,*tub-Gl80*^*ts*^*/UAS-miR-210/+*) restricted only during development (29°C-23°C), or only during adulthood (23°C-29°C), with respect to positive (constitutive over-expression, *tim-Gl4(U)/UAS-miR-210*) and negative (*tim-Gl4(U)/+*) controls. Over-expression was modulated by using the Gal4-Gal80^ts^-UAS ternary system. Mean ± SEM. Mann-Whitney U test: *** p<0.005, **p<0.01; ns: not significant. (Dark grey bar: ZT3, light grey bar: ZT15).

In contrast to the l-LNvs, s-LNvs projections were able to reach the dorsal part of the brain, the area in which they usually send their axons. Since their termini experience daily circadian changes in morphology, shifting from the defasciculated (ZT3) to the fasciculated (ZT15) state of their termini [51], the arborisations of these distal projections were investigated at ZT3 and ZT15, quantifying the number of axon crosses [51]. As reported in Fig 8A and 8B, while *miR-210* over-expression (*tim-Gl4(U)/UAS-miR-210*) reduced the number of axonal crosses (blocking the termini in the fasciculated state), the complete absence of *miR-210* (*yw(Ti-Gal4)miR-210*^*KO*^*)* weakened the cycling of axonal crosses number (Fig 8A and 8B). Taken together, these results implicate *miR-210* in the remodelling of PDF positive neuron morphology.

### *miR-210* up-regulation during development affects l-LNvs morphology and Lights-ON anticipation but not circadian rhythmicity in DD

To ascertain if the arrhythmicity of flies over-expressing *miR-210* was due to developmental defects, we took advantage of the Gal4-Gal80^ts^-UAS ternary system to target and manipulate the spatial and temporal expression of *miR-210* in TIM-expressing cells. *tim(UAS)Gal4*,*tub-Gal80*^*ts*^*/UAS-miR-210* flies (hereafter *tim-Gl4(U)*,*tub-Gl80*^*ts*^*/UAS-miR-210*) were analysed at both restrictive (29°C) and permissive (23°C) temperatures.

**Fig 9:**
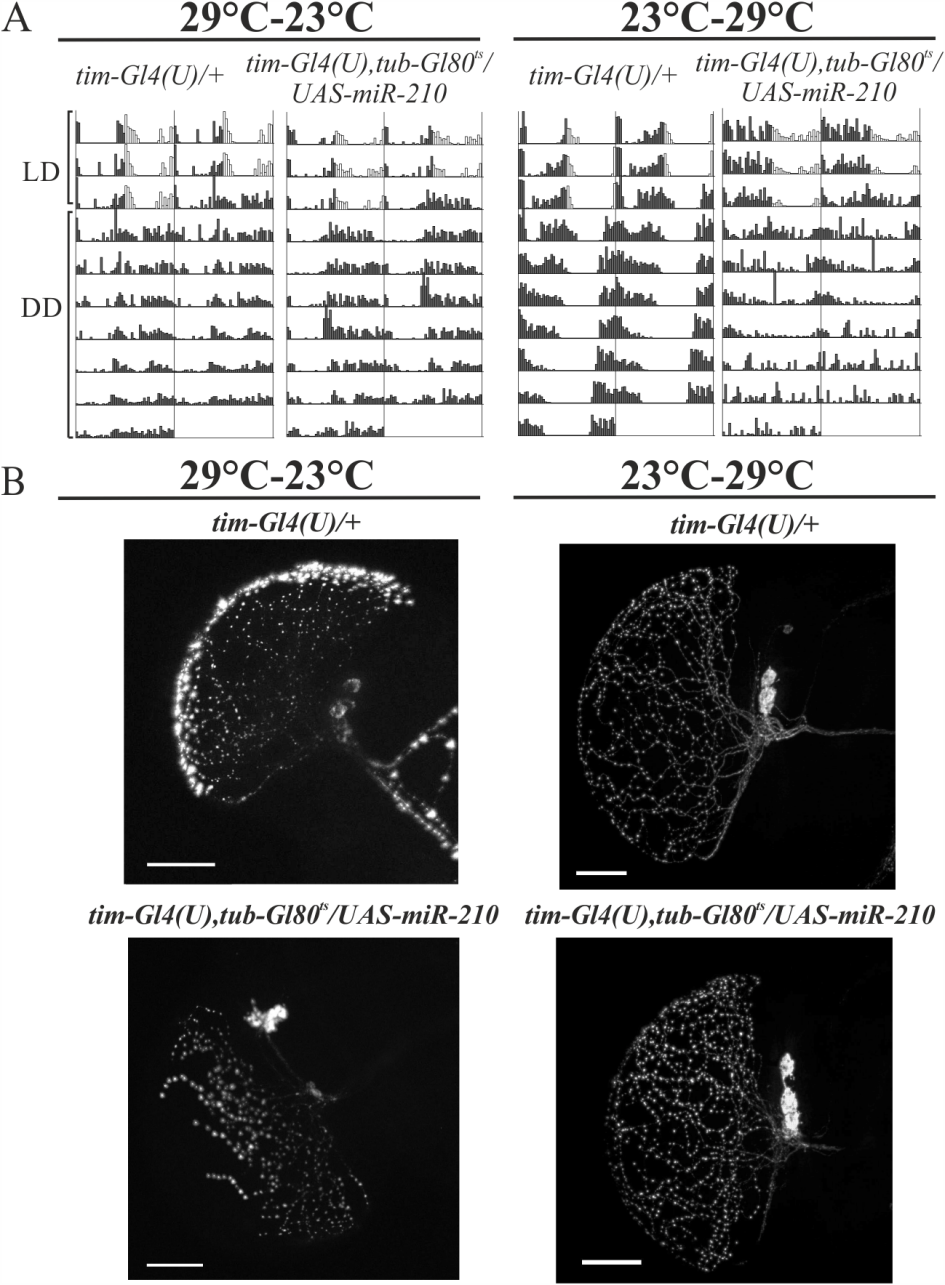
Temporal expression of miR-210 in clock cells. (A) Double-plotted locomotor activity of a single representative fly per genotype, recorded for 3 complete days under LD and for 7 complete days under DD. The indicated temperatures refer to *tim-Gl4(U)*,*tub-Gl80*^*ts*^*/UAS-miR-210* flies and controls (*tim-Gl4U)/+*) developed at 29°C and analysed at 23°C (29°C-23°C) or developed at 23°C and analysed at 29°C (23°C-29°C). (dark grey: dark phase; white: light phase). (B) Confocal stack images representing l-LNvs arborisation patterns in flies over-expressing *miR-210* during different temporal transects (see above). Whole brains were collected at ZT0 and PDF was detected. Scale bar: 25 μm.

When *miR-210* expression was activated only during development (29°C-23°C) flies were mostly rhythmic under DD conditions (Fig 9A, Table 4), and displayed normal PER oscillations in LD (S6A Fig), as well as normal circadian changes in the morphology of s-LNv dorsal termini (Fig 8C). However, the number of PDF vesicles and the arborisation of the l-LNvs were affected (Figs 7B and 9B) and most of the flies lost their ability to anticipate the Lights-ON transition (Table 4).

**Table 4:** Locomotor activity of flies with temporal control of *miR-210* over-expression.

Genotype	N° tot	N° Alive	N° R	% R	τ		SEM	% MA	% EA
**29°C—23°C**									
*w;tim(UAS)-Gal4*,*tub-Gal80*^*ts*^*/UAS-miR-210*	95	91	62	68.13	24.18	±	0.07	**29.67**	54.95
*w;tim(UAS)-Gal4*,*tub-Gal80*^*ts*^*/+*	94	91	70	76.92	24.09	±	0.05	62.64	75.82
*w;tim(UAS)-Gal4/+*	91	85	75	88.24	23.72	±	0.06	87.06	90.59
*w;UAS-miR-210/+*	92	89	80	89.98	23.89	±	0.05	86.52	91.01
*w;tim(UAS)-Gal4/UAS-miR-210*	93	82	7	**8.54**	24.52	±	0.16	**47.56**	79.27
**23°C—29°C**									
*w;tim(UAS)-Gal4*,*tub-Gal80*^*ts*^*/UAS-miR-210*	61	14	7	**50.00**	24.10	±	0.23	**42.86**	78.57
*w;tim(UAS)-Gal4*,*tub-Gal80*^*ts*^*/+*	63	51	49	96.08	23.68	±	0.07	98.04	86.27
*w;tim(UAS)-Gal4/+*	59	49	49	100.00	23.34	±	0.04	67.35	93.88
*w;UAS-miR-210/+*	30	12	11	91.67	23.69	±	0.16	100.00	100.00
*w;tim(UAS)-Gal4/UAS-miR-210*	59	25	6	**24.00**	24.17	±	0.25	**28.00**	68.00

Male flies were monitored for three days in LD 12:12 and seven days in DD. Flies with the following genotypes: *w;tim(UAS)-Gal4*,*tub-Gal80*^*ts*^*/+*, *w;tim(UAS)-Gal4/+* and *w;UAS-miR-210/+* flies are negative controls, while *w;tim(UAS)-Gal4/UAS-miR-210* flies are positive controls. *miR-210* over-expression (*w;tim(UAS)-Gal4*,*tub-Gal80*^*ts*^*/UAS-miR-210*) was activated only during development (29°C-23°C) or only during adulthood (23°C-29°C). Period values (τ) were averaged over all rhythmic flies per genotype. R: rhythmic flies; N: number of tested flies. MA (morning anticipation) and EA (evening anticipation) were detected, fly-by-fly, examining the bout of activity prior to light transitions as in [45].

In contrast, when *miR-210* expression was activated only during the adult stage (23°C-29°C), flies showed a high mortality possibly reflecting the increased level of *miR-210* expression at this temperature. The few flies that remained alive were weakly rhythmic over the first days and then became arrhythmic and again did not anticipate Lights-ON (Fig 9A, Table 4). PER oscillations were normal compared to those of controls (S6A Fig). The l-LNvs did not exhibit any abnormal arborisations or shape or reduced number of PDF vesicles nor was the s-LNvs termini structural plasticity compromised (Figs 7B, 8C and 9B).

To assess the functional performance of the large LNvs, sleep was quantified in flies with or without these neuronal defects. Loss of PDF arborisation significantly increased daytime sleep (S6B Fig). These data, which are consistent with those of Sheeba et *al*. and Shang et *al*. [52,53], indicated that *miR-210* affects total sleep levels and its temporal distribution.

### *miR-210* regulates multiple genes involved in different pathways

**Fig 10:**
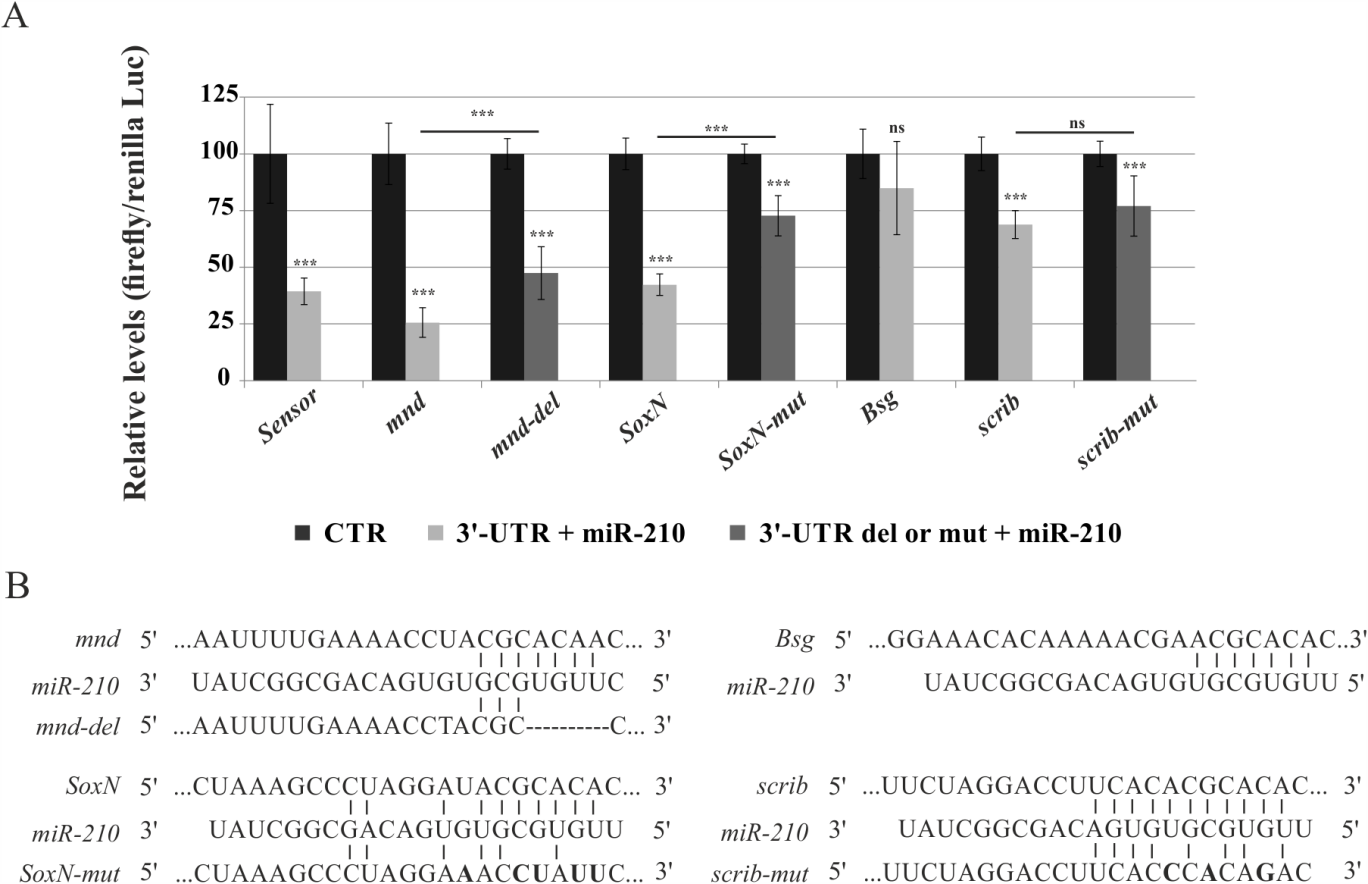
*miR-210* modulates the expression of multiple genes. Luciferase assays were performed 24 hours post-transfection on S2R+ cells transfected with miR-210 precursor (*pUAST-miR-210* + *p-Act-Gal4*) or negative controls (only *p-Act-Gal4*). (A) Luciferase relative levels in cells transfected with reporter constructs containing wild-type or mutant 3’-UTRs for the indicated genes, or a synthetic sequence including 3 perfect *miR-210* binding sites (sensor). Results from three independent experiments are shown as mean ± SD of firefly luciferase activity relative to controls. t test; *p<0.05; **p<0.01; ***p<0.005; ns, not significant. (B) Reporter constructs containing miR-210 wild type or mutant binding sites in *mnd*, *SoxN*, *Bsg*, *scrib* and 3’-UTRs (sensor). mut, mutation; del, deletion.

To identify genes directly or indirectly regulated by *miR-210*, gene expression signatures of adult fly brains were defined and sampled at ZT0, by over-expressing *miR-210* in the TIM-expressing cells (*tim-Gl4(U)/UAS-miR-210*). The results were compared to their control (*tim-Gl4(U)*). Significance Analysis of Microarray (SAM)-two-class analysis identified 2,376 differentially expressed genes (941 up-regulated and 1,435 down-regulated), considering a 7.0% false discovery rate (S3 Table). A total of 78 genes were identified by comparing these results with putative *miR-210* targets obtained from two target prediction algorithms, mirSVR and TargetScan 6.2 [23,54–56] (S4 Table). The probability of obtaining this enrichment by chance is *P* = 3.774758 e^-15^, as calculated from the hypergeometric distribution. Two genes within the top-ranking *miR-210* target candidates (the most down regulated, *echinus* and *minidisc*, S4 Table), and one chosen by a literature search (*SoxNeuro*, for its role in Central Nervous System development [57,58]) were selected and qRT-PCR analysis was used to validate their expression (S7 Fig). The functional annotation tool DAVID, [59] was used to analyse the 2,376 differentially expressed genes to identify the biological processes represented in the expression signatures. A consistent number of genes involved in “Neuron development” (GO:0048666, 54 genes, BH adjusted p-value: 0.096, S5 Table), “Circadian rhythms” (GO:0007623, 22 genes, BH adjusted p-value: 0.095, S6 Table) and “small GTPase mediated signal transduction” (GO:0007264, 19 genes, BH adjusted p-value: 0.098, S7 Table) were identified. A panel of 4 putative targets, already detected to be associated *in vivo* to the AGO1 in fly heads and therefore miRNAs regulated [37], *SoxNeuro* (*SoxN*), *minidiscs* (*mnd*), *Basigin* (*Bsg*) and *scribbled* (*scrib*), belonging to the biological processes mentioned above, were selected for a luciferase assay to test for a direct interaction (Fig 10A). *mnd* was selected based on the ranking value (S4 Table). For all other genes, selection was based on a literature search, for their involvement in axon guidance (*SoxNeuro* [58]), or cell morphogenesis (*scribbled* [60–62] and *Basigin* [63]). Among these, *miR-210* targets were identified by performing luciferase assays in *miR-210* over-expressing or control S2R+ Drosophila cells, transfected with reporter vectors containing wild-type or mutated 3’-UTRs. A significant reduction in luciferase activity was observed in cells transfected with the vectors containing wild-type 3’-UTRs of the *mnd*, *SoxN* and *scrib* putative targets, with the exception of *Bsg*. Normal levels of luciferase activity were restored in cells transfected with the vectors containing mutated 3’-UTRs of *SoxN* and *mnd*, with the exception of *scrib* (Fig 10A and 10B). Therefore, *mnd*, and *SoxN*, may be direct targets of *miR-210*.

An *in vivo* RNAi screening was also performed on these genes, in order to determine whether their down-regulation in the TIM-expressing cells was able to phenocopy the loss of rhythmicity observed in flies over-expressing *miR-210*. We set out to analyse RNAi lines for the two genes mentioned above crossed with the *tim-Gl4(U)* line. Males from the progeny were tested for 3 days under LD, and for 7 days under DD. A qRT-PCR was performed in adult fly heads in order to validate the RNAi lines (S8 Fig). Down-regulation of the genes examined did not affect morning or evening anticipation of light transitions nor rhythmicity under DD (Table 5). A whole mount PDF staining of the brain was performed at ZT0 to examine the morphology of the PDF projections of l-LNvs in flies knocked down for *mnd* and *SoxN*. No aberrant projections were detected in the *tim-Gal4/UAS-RNAi* flies analysed (S9 Fig).

**Table 5:** Locomotor activity of *mnd* and *SoxN* knock down flies.

Genotype	N° Tot	N° Alive	N° R	% R	τ		SEM	% MA	% EA
*w;tim(UAS)-Gal4/+;UAS-RNAi-mnd/+*	32	31	23	74.19	23.99	±	0.08	93.55	100
*w;;UAS-RNAi-mnd/+*	30	30	29	96.67	23.62	±	0.06	93.33	100
*w;tim(UAS)-Gal4/+;UAS-RNAi-SoxN/+*	62	61	39	63.93	23.96	±	0.05	75.41	98.36
*w;;UAS-RNAi-SoxN/+*	60	53	49	92.45	23.83	±	0.06	77.36	96.23

The progeny of each RNAi line (*w;;UAS-RNAi-mnd/+* and *w;;UAS-RNAi-SoxN/+*) crossed with the *tim-Gal4* driver were analyzed for 3 days under LD and 7 days under DD. R: rhythmic flies. Period values (τ) were averaged over all rhythmic flies per genotype and compared to those of controls (RNAi/+ lines). MA (morning anticipation) and EA (evening anticipation) were detected, fly-by-fly, examining the bout of activity prior to light transitions as in [45].

### *miR-210* affects the morphology of *Drosophila* neuronal cells

**Fig 11:**
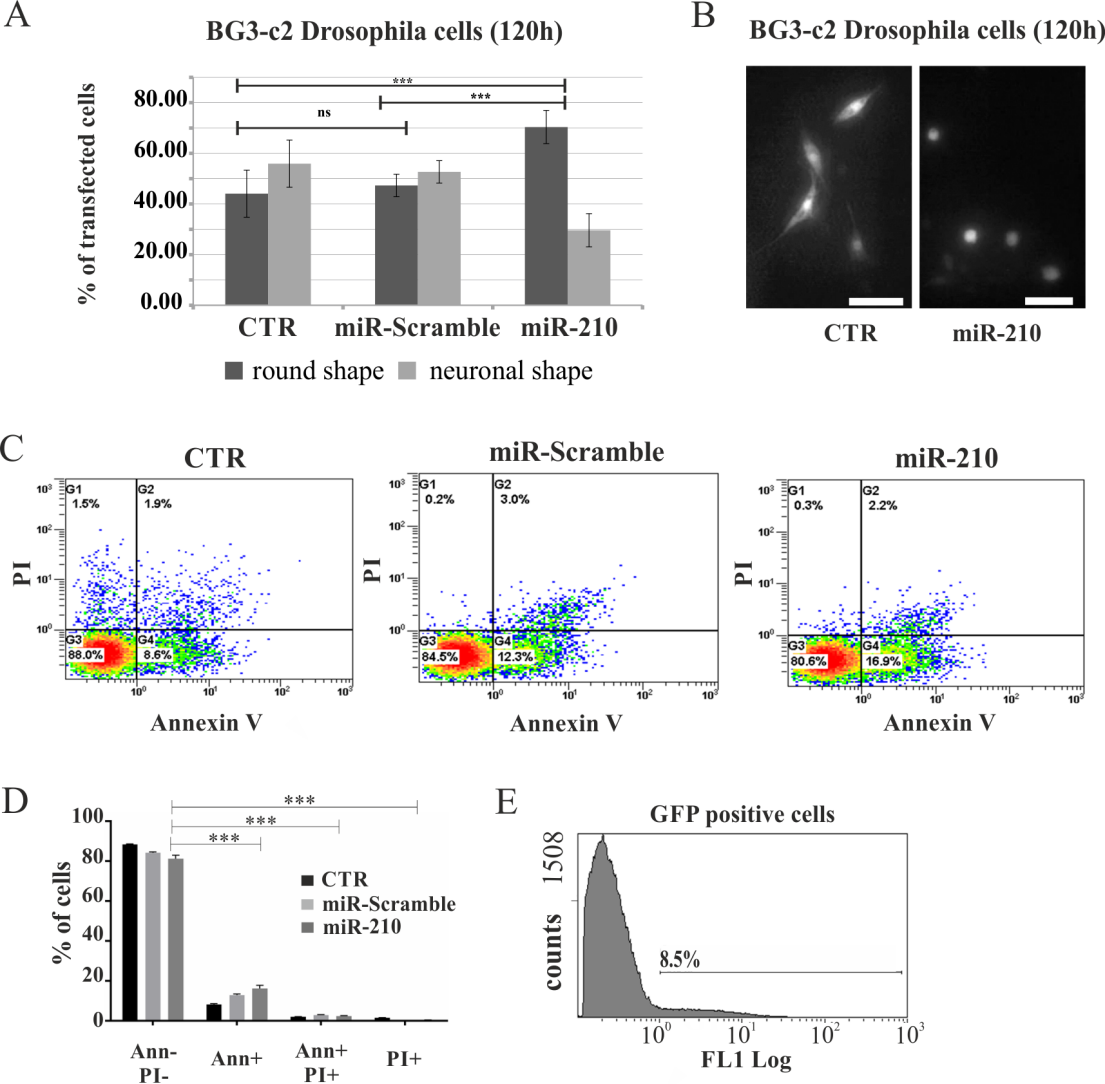
*miR-210* over-expression in Drosophila BG3-c2 neuronal cells. (A) BG3-c2 Drosophila neuronal cells were co-transfected with *pUAST-miR-210*, *p-Act-Gal4* and *p-Act5c-stable-neo-GFP (miR-210)* or *pUAST-miR-Scramble*, *p-Act-Gal4* and *p-Act5c-stable-neo-GFP (miR-Scramble)*. In addition, co-transfaction with *p-Act-Gal4* and *p-Act5c-stable-neo-GFP* was used as a control (CTR). (A) Histograms show the percentage of transfected cells (GFP-positive), measured after 120 hours from transfection, with round or neuronal shapes. t test: ***p<0.005, ns: not significant. (B) GFP expression in BG3-c2 Drosophila cells transfected with miR-210 (right panel) and control (left panel) after 120 hours (see above for details). It should be noted that most of the neurons treated with miR-210 exhibited a round shape. Scale bar 36 μm. (C) Representative plots of BG3-c2 GFP positive cells (CTR, *miR-Scramble* and *miR-210*) 120 hours post-transfection and double-stained with Annexin V and propidium iodide (PI). (D) The histogram shows the higher percentage of GFP positive live cells (Annexin V-/PI-), with respect to early apoptotic (Annexin V+), or late apoptotic/necrotic cells (Annexin V+/PI+ and PI+, t test *** p<0.001) of all treated samples. (E) BG3-c2 transfection efficiency.

To further examine any *miR-210* role in shaping neuronal projection patterns, a transient transfection of *miR-210* was performed in *Drosophila* neuronal BG3-c2 cells, which, once plated, spread arborisations in ~55% of neurons. While treated cells were co-transfected with a *pAct-GFP*, *pAct-Gal4* and *pUAST-miR-210* plasmids, the controls were co-transfected with *i) pAct-GFP* and *pAct-Gal4*, or *ii) pAct-GFP*, *pAct-Gal4* and *pUAST-miR-Scramble* plasmids. GFP-positive cells were then analysed after 120 h. Approximately 70% of *miR-210* treated cells were found to lose their arborizations, compared to 45% in controls (Fig 11A and 11B). No differences were observed compared to controls when a *miR-Scramble* was used for transfection (Fig 11A). A propidium iodide test and Annexin V Apoptosis Detection Test were performed by cytofluorimetry to exclude the possibility that the morphological features of the BG3-c2 Drosophila cells were a result of a necrosis due to environmental perturbation (over-expression of *miR-210*), or to a programmed cell death triggered by the miRNA itself (Fig 11C and 11D). Less than 9% of cells transfected for *miR-210* (among the GFP positive) were shown to be undergoing apoptosis (Annexin-positive) and no neuronal necrosis was detected with a transfection efficiency of 8.5% (Fig 11E). These results support the view that *miR-210* over-expression plays a role in preventing arborisations *in vitro*, reflecting the similar observation with l-LNvs in the OL *in vivo*.

### *miR-210* over-expression during development induced visual defects

**Fig 12:**
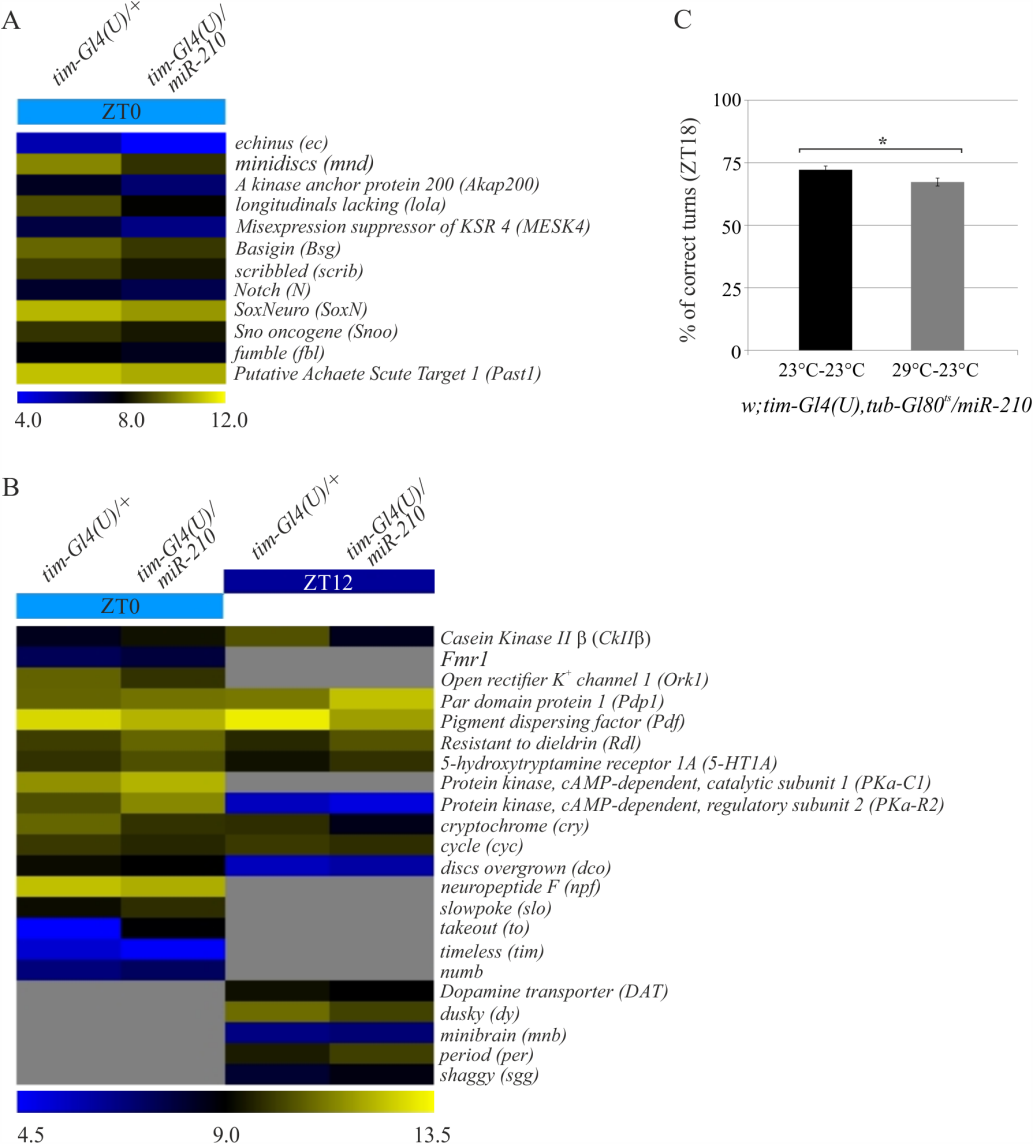
Altered gene pathways in *miR-210* over-expressing flies. Heat maps representing a selection of deregulated transcripts, provided by the DAVID tool, in *tim-Gl4(U)/UAS-miR-210 versus* control samples (*tim-Gl4(U)/+*) involved in (A) phototransduction (12 transcripts). A color-coded scale for the normalized expression values is used: yellow and blue represent high and low expression levels in *miR-210* over-expressing flies, compared to controls. The expression level of each transcript was calculated as Log2 (*miR-210*/CTRL). A complete list of differentially expressed genes identified by SAM two class algorithm is provided in the Supplementary Information (S3 and S9 Tables). (B) Heat map representing deregulated transcripts, provided by the DAVID tool, in *tim-Gl4(U)/UAS-miR-210* versus control samples (*tim-Gl4(U)/+*) involved in circadian rhythms (22 transcripts), at ZT0 and ZT12. (C) Optomotor responses at ZT18 of *tim-Gl4*,*tub-Gl80*^*ts*^*/UAS-miR-210* flies kept at 23°C (23°C-23°C) or 29°C (29°C-23°C) during development. A total of 100 flies per genotype were analysed. *miR-210* over-expressing flies (grey) showed a significant decrease in the optomotor response compared to controls (black). (Mean ± SEM; t-test * p<0.05).

As demonstrated above, over-expression of *miR-210* in TIM-expressing cells during development causes an irreversible disruption of the l-LNvs distal arborisation in the optic lobe, and an aberrant neuronal cell body shape. Gene expression analysis highlighted the presence of genes that were down-regulated by *miR-210* over-expression. Among these, genes involved in the organization and development of photoreceptors were also identified (Fig 12A, S8 Table). It was therefore decided to use the optomotor response to evaluate the visual ability of flies in which *miR-210* was over-expressed only during pre-adulthood developmental stages. *tim-Gl4(U)*,*tub-Gl80*^*ts*^*/UAS-miR-210* flies were raised at a restrictive (29°C-23°C) or permissive (23°C-23°C) temperature to over-express or prevent the expression of *miR-210* during development, respectively. Adult males were placed in an incubator at 23°C for 3 days and collected and analysed at ZT18, when wild type flies usually perform best [64]. Flies over-expressing *miR-210* during development gave significantly fewer correct responses compared to controls (Fig 12C). We concluded that over-expression of *miR-210* during development induces visual defects. This is consistent with our microarray analysis, indicating an enrichment of affected genes involved in the photoreception pathways.

### Circadian genes altered by *miR-210* over-expression

A cluster of genes involved in maintaining circadian rhythmicity was identified amongst those that were differentially expressed (down regulated) at ZT0 in *tim-Gl4(U)/UAS-miR-210* flies compared to controls: *tim*, *pdf*, *cryptochrome* (*cry*), *open rectifier k*^*+*^ *channel 1* (*ork1*), *cyc*, *neuropeptide F* (*npf*), *dco* and *pdp1* (Fig 12B, S6 Table). An additional gene expression analysis was performed at ZT12, in both the over-expressing flies and controls (S9 Table). *pdf*, *cry* and *cyc* transcripts were down-regulated in *miR-210* over-expressing flies at both ZT0 and ZT12, while *sgg*, *per*, *disc overgrown* (*dco-* also called *dbt*) and *pdp1* were up-regulated at ZT12 (Fig 12B). Only *dbt* was identified *in silico* as a putative target for *miR-210* (S4 Table), but it has not been identified as a miRNA-regulated transcript *in vivo* [37]. Our findings suggest that *miR-210* modulates the expression of circadian clock components mostly indirectly.

## Discussion

In recent years, it has emerged that miRNAs play important roles in modulating a variety of physiological process. This study focuses on *miR-210* which in mammals is involved in processes such as angiogenesis, neurogenesis, mitochondrial metabolism, apoptosis, proliferation and hypoxia [65]. In Drosophila the functional role of *miR-210* has not been fully characterized.

Expression levels of *miR-210* have been shown to increase in *cyc*^*01*^ mutant flies [43], and are cycling under LD in PDF neurons [44], suggesting a link between *miR-210* and the regulation of the circadian clock machinery. We showed that *miR-210* is a modulator of the circadian locomotor activity of flies, under both LD and DD conditions because overexpression and knock-out of *miR-210* in clock cells significantly delayed or advanced, respectively, the phase of the evening onset of flies under light-dark cycles, without affecting PER cycling in canonical clock neurons. This suggests that *miR-210* regulates genes involved in the control of the circadian clock output pathways. In addition, *miR-210* up-regulation in clock cells rendered most of the flies arrhythmic in DD (and phase delayed the remaining rhythmic individuals), while the knock-out significantly phase advanced locomotor activity.

In a recent paper, Chen and Rosbash identified *miR-210* as one of the miRNAs expressed in PDF positive neurons, cycling with a peak in the middle of the day (ZT6), as measured by qRT-PCR [44]. Here we report its expression pattern in photoreceptors, Optic Lobe, Antenna Lobe, Mushroom Bodies and H-B eyelet, but we failed to detect *miR-210* expression in clock neurons, as measured by a GFP reporter. A potential explanation for this is that we were not able to detect GFP in clock neurons by confocal microscopy due to limitations of this technique compared to qRT-PCR. Our behavioural data however, supported the hypothesis that *miR-210* is transcribed in PDF clock neurons. By manipulating *miR-210* expression levels with Gal4-Gal80-UAS, we were able to unveil the anatomical contribution of different clusters of clock cells in defining the locomotor activity phase of flies. We found that *miR-210* over-expression in PDF positive neurons is sufficient to delay the evening onset of flies under LD and, surprisingly, its expression in these neurons is necessary to ensure the correct phase under DD. Although these results corroborate the data previously published by Chen and Rosbash [44], we could not completely exclude that *miR-210* might be also released from the H-B eyelet, via synaptic transmission, targeting the small LNvs. In the presence of light, *miR-210* could be released by the H-B eyelet, to accumulate in PDF expressing neurons, with a peak of expression in the middle of the day at ZT6, as reported by Chen and Rosbash [44]. We formulate this hypothesis because flies delayed their activity under LD, when the amount of *miR-210* was highly elevated in PDF positive neurons, due to its constitutive expression driven by the Gal4-UAS system. On the other hand, weak or no advance in evening phase was observed in LD when *miR-210* transcription was repressed in PDF/CRY positive neurons, despite the dramatically advanced phase activity under LD observed in the *miR-210* knock out. This suggests that other structures expressing *miRNA-210* (i.e. the HB-eyelet) may contribute to modulating evening activity. Altogether, these data suggest that *miR-210* expression is required in the OL, AL, MB, photoreceptor and H-B eyelet to define the normal evening activity onset of flies when in LD.

By contrast, under DD, *miR-210* seems to be required at least in the PDF positive neurons as both repression of *miR-210* expression in PDF positive neurons or *miR-210* knock out show a 4–6 h advanced activity phase. We also observed that *miR-210* up-regulation in TIM- or CRY*-* expressing cells or *miR-210* depletion in CRY expressing cells, impinge on the circadian rhythmicity of flies under DD. This also suggests that an unbalanced *miR-210* transcription between clock neurons and photoreceptors, H-B eyelet, MB and AL, may affect the clock’s circadian output reinforcing the view that *miR-210* levels are critical for the circadian activity output of flies.

*miR-210* also controls the morphogenesis and the structural plasticity of the PDF expressing neurons. In the present study, the up-regulation of *miR-210* levels in clock cells affected the l-LNv body shape and resulted in aborted termini of their neurites in the distal part of the OL. Although this phenotype was not severe in all the brains analysed, all were affected to some extent. Day-time sleep was increased in flies with aberrant projections in the PDF positive l-LNvs compared to control, suggesting that the function of these cells is impaired [52,53]. *miR-210* over-expression during development results in aberrant projections of the large LNvs which appear during the mid-stage of the fly metamorphosis [7]. In addition, the LD and DD behaviour of flies with aberrant large PDF projections suggests their morphogenetic defects do not interfere with circadian locomotor activity.

By contrast, the s-LNv neuronal pattern of *tim-Gal4/UAS-miR-210* over-expressing flies did not appear to be affected: their projections still reached the dorsal part of the brain. However, it is well established that s-LNvs termini undergo circadian plastic changes in their morphology, with a higher degree of arborisation in the morning (defasciculated state) and a lower degree in the evening (fasciculated state) [51]. In between, at ZT6, *miR-210* reaches its maximum expression in the PDF positive cells [44]. We observed that in *miR-210* KO, the cycling between the defasciculated and fasciculated state of the small-LNvs dorsal projections was damped as when *miR-210* is constitutively over-expressed with the *tim*-promoter. It is interesting to highlight that the restricted temporal over-expression of *miR-210* (only during developmental or adulthood) did not affect the s-LNvs terminal plasticity, suggesting that the s-LNvs phenotype of *tim-Gal4/UAS-miR-210* flies is likely to be due to developmental defects caused by *miR-210* up-regulation, coupled with its higher levels in clock cells during adulthood. Similarly, only when over-expression was maintained through development and adulthood (*tim-Gal4/UAS-miR-210* flies) PER and *per* transcript levels were significantly higher compared to controls in all clock neurons. This implicates *miR-210* in the modulation of the expression of developmental genes, as well as genes that affect expression of components of the core molecular clock.

Further analysis of *Drosophila* small RNA expression datasets revealed that *miR-210* represents more than 1% of all miRNAs in heads [66]. This places *miR-210* in the top 50 abundant miRNAs expressed in the brain [67], highlighting its importance in regulating biological processes in this tissue. As *miR-210* is one of the top ten miRNAs that are predicted to be major regulators of developmental genes [68], a microarray analysis of adult fly brains was performed at ZT0. This was to examine its biological function and to identify the most likely targets. Analysis of the down-regulated transcripts in *miR-210* over-expressing flies revealed an enrichment of genes involved in neuronal development including *SoxNeuro*, *longitudinal lacking* (*lola*) and *Notch* together with putative targets of *miR-210*. All these genes are involved in the axonal patterning processes and they participate in photoreceptor differentiation. *lola*, encoding a transcriptional factor, was found to be a target of *SoxNeuro* [58] and *lola* expression in the Drosophila eye disc is activated or repressed by Epidermal growth factor receptor and Notch, respectively, to determine R3, R4 and R7 photoreceptor cell fate [69]. *SoxNeuro* was shown to be a direct target of *miR-210 in vitro* but the down-regulation of *SoxN* did not affect the development of PDF arborisations in the OL. None of the down-regulated genes that are also putative targets of *miR-210* affected the PDF projection or perikaria of the l-LNvs. As l-LNvs control relevant physiological functions such as setting the activity of flies and mediating light-arousal and sleep [6,52,53,70], their altered development due to *miR-210* over-expression might depend on the simultaneous perturbation of the expression levels of several genes.

We also expressed *miR-210* in the BG3-c2 neuronal cell line derived from the Drosophila larval CNS. This particular cell line has been reported to express the *pdf* transcript [71] and is also characterized by neurons mostly developing long finger-like arborisations, a few days after they are plated. This makes them a suitable *in vitro* system to investigate the role of *miR-210* in modulating neuronal cells morphology. Interestingly, a significant fraction of the BG3-c2 cells expressing *miR-210* lost their arborisations compared to their controls, paralleling the effects on the arborisation we reported *in vivo* in the l-LNvs.

*miR-210* is highly conserved between humans and flies [72]. Up- or down-regulation of hsa*-miR-210* expression levels has already been associated with varying human diseases [65]. Moreover, it has been reported that *miR-210* is up-regulated in a murine model of oxygen-induced proliferative retinopathy (OIR) [73]. This is interesting as we have shown that *miR-210* over-expression during development alters the flies’ ability to perceive motion. It is not currently known, however, if this impairment is due to aborted projections of the l-LNvs in the optic lobe, or to a developmental defect of photoreceptors (i.e. the R3 and R4, the fate of which depends upon *SoxN*, *lola*, *Egfr* and *N* interactions) [69].

To conclude, our *in vivo* and *in vitro* data indicate that Drosophila *miR-210* affects behavioural circadian rhythmicity and the morphology of the PDF positive LNvs. It may also affect light signalling from the visual system to clock neurons and, in turn, the circadian phase of locomotor activity.

## Methods

### Drosophila strains

Flies were raised on standard cornmeal-yeast agar food in LD 12:12 cycles at 23C°. Several independent UAS-miR-210 lines were generated by cloning the 153 bp of the genomic region that contained the *pre-miR-210* in a pUAST plasmid [74]. The following primers were used: *F*: *GTAGTGATTCACCGACCACGT*, *R*: *ACCACGATGATGGAACAATG*. Two of these lines (*UAS-miRNA-210*.*5* and *UAS-miRNA-210*.*9*) were crossed to *tim-Gal4(UAS)* and the characterization of the progeny for behavioural (locomotor activity profiles and optomotor response, S10 Table and S10 Fig) and molecular features (PER and PDF expression pattern and miRNA-210 expression levels, S10 Fig) gave similar preliminary results. The *UAS-miRNA-210*.*9* was therefore selected for subsequent analyses and named *UAS-miRNA-210*. The other strains used in this study were previously characterized: *tim-Gal4(UAS)* [75], *pdf-Gal4* [4], *C929-Gal4* [48], *tub-Gal80*^*ts*^ [76], *cry-Gal4* [77], *gal1118-Gal4* [47], *pdf-Gal80* and *cry-Gal80* [9] The RNAi lines were obtained from the Vienna Drosophila RNAi Center [78]. The *RNAi-SoxNeuro*, the *yw(Ti-Gal4)miR-2l0*^*KO*^, and the *yellow*^*1*^ strains, were obtained from the Bloomington Drosophila Stock Center. Controls (Gal4 and UAS strains) and mutant flies (*yw(Ti-Gal4)miR-210*^*KO*^ and *yellow*^*1*^) were crossed with *w*^*1118*^ males prior to analysis.

### Circadian behaviour

The locomotor activity of 1 to 3 day-old males was recorded for 3 days in LD and 7 days in DD conditions at 23C° or 29C°, using the Drosophila Activity Monitor System (Trikinetic). Data were collected every 5 min and then analysed in 30 min bins using spectral analysis and autocorrelation, as described elsewhere [79]. Morning and evening anticipations were detected fly-by-fly, by examining the mean activity over 3 days under LD conditions and in accordance with [45]. The Morning Index was calculated as in [80]. Three days of activity in LD were used to generate average activity bar graphs. The LD evening phase onset was calculated manually on these graphs, as described in [45]. In particular, the evening phase onset was considered to be present when a bout of activity occurred after a period of rest during the day but it had to be composed of continuous movement with no more than one zero activity bin interspersed within, and with a steady increase in activity levels defining the onset. The DD activity phase was calculated manually, as the highest bout of activity occurring during the fourth day of constant conditions on smoothed data [79]. Sleep amount was calculated from the locomotor activity data by using a Microsoft Excel script in which sleep was defined as 5 min of consecutive inactivity of the flies [81].

### Total RNA isolation

1 to 3 day-old male flies were entrained for at least 3 complete days and then collected at the indicated time points. Total RNA was extracted from approximately 25 brains for each genotype using ZR RNA MicroPrep (ZYMO RESEARCH), according to the manufacturer’s instructions, and then quantified using the ND-1000 spectrophotometer (Nanodrop, Wilmington, DE). The quality of RNA was checked by capillary electrophoresis (RNA 6000 Nano LabChip, Agilent Bioanalyzer 2100, Agilent Technologies) and only samples with RNA Integrity Number (R.I.N.) values > 6 were used for microarray analysis. Where appropriate, total RNA was isolated from 30 male fly heads using Trizol (Life Technologies), according to the manufacturer’s instructions.

### Microarray labelling and hybridization

Gene expression profiling was carried out on the controls (*w;tim-Gl4(U)/+*) and miR-210 over-expressing flies (*w;tim-Gl4(U)/miR-210*), sampled at ZT0 and ZT12, using the Drosophila 2.0 custom platform (GPL18767). Four biological replicates were analysed for the controls and miR-210 over-expressing flies, for a total of 8 microarray experiments. Fifty ng of total RNA was labelled with “Low Input Quick Amp Labeling Kit, one color” (Agilent Technologies, CA), following the manufacturer’s instructions. The synthesized cDNA was transcribed into cRNA, labelled with Cy3-dCTP and purified with RNeasy Mini columns (Qiagen, Valecia, CA). The quality of each cRNA sample was verified by the total yield and specificity calculated with NanoDrop ND-1000 spectrophotometer measurements (Nanodrop, Wilmington, DE). Six hundred ng of labelled cRNA were used in each reaction and the hybridization was carried out at 65°C for 17 hours in a hybridization oven-rotator (Agilent Technologies, Palo Alto, CA). The arrays were washed using “Agilent Gene expression washing buffers” and “Stabilization and Drying Solution,” as recommended by the supplier. Slides were scanned on an Agilent microarray scanner (model G2565CA), and the Agilent Feature Extraction software version 10.5.1.1 was used for image analysis. Gene expression data are available in the GEO database using the accession number GSE77245.

### Statistical analysis of gene expression data

Inter-array normalization of the expression levels was performed using the quantile method to correct experimental distortions [82]. A normalization function was applied to the expression data of all the experiments and the values of within-array replicate spots were averaged. Feature Extraction Software, which provided spot quality measures, was used to evaluate the quality and reliability of the hybridization. In particular, the flag “glsPosAndSignif” (set to 1 if the spot had an intensity value that was significantly different from the local background and to 0 in all other cases) was used to filter out unreliable probes: a flag = 0 was marked as “not available (NA)”. Probes with a high proportion of “NA” values were removed from the dataset to attain a more solid, unbiased statistical analysis. Fifty percent of “NA” was used as the threshold in the filtering process, and about 23,700 *Drosophila* transcripts were obtained. Cluster analysis and profile similarity searches were performed with Multi Experiment Viewer version 4.8.1 (tMev) of the TM4 Microarray Software Suite. The identification of differentially expressed genes was performed using two class Significance Analysis of Microarray (SAM) algorithm [83] with default settings. SAM uses a permutation-based multiple testing algorithm and identifies significant genes and miRNA with variable false discovery rates (FDR). This can be manually adjusted to include a reasonable number of candidate genes with acceptable and well-defined error probabilities. The normalized expression values of the biological replicates for each genotype were log2-transformed and averaged. The list of differentially expressed genes was functionally classified using the DAVID Gene Functional Classification tool (https://david.ncifcrf.gov/, [59]) to identify significantly enriched biological processes (Modified Fisher Exact p-value < 0.05).

### miRNA target prediction

The TargetScan 6.2 (http://www.targetscan, Release: June 2012, [23] and mirSVR (http://www.microrna.org, Release: August 2010, [54]) algorithms were used to predict dme-miR-210 targets. To identify the most likely targets, attention was focused on putative mRNAs differentially expressed in miR-210 overexpressing flies, sampled at ZT0 and ZT12 [55,56].

### Quantitative reverse transcriptase real-time PCR

Twenty-five brains were dissected for miR-210-3p and 2S rRNA quantification assays at the indicated time points. Each RT reaction (15 μl) contained 10 ng of total purified RNA, 5X stem-loops RT primer, 1X RT buffer, 0.25 mM each of dNTPs, 50U MultiScribe reverse transcriptase and 3.8 U RNAse inhibitor. The reactions were incubated in a thermocycler (Applied Biosystems) in 0.2 ml PCR tubes for 30 min at 16°C, 30 min at 42°C, followed by 5 min at 85°C, and then kept at 4°C. The resulting cDNA was quantitatively amplified in 40 cycles on an ABI 7500 Real-Time PCR System, using TaqMan 2XUniversal Master Mix no AmpErase UNG Mix and TaqMan MicroRNA Assays (Assay ID for miR-210-3p: 005997 and Assay ID for 2S rRNA: 001766 Applied Biosystems). Three replicates of each sample and endogenous control were amplified for each real-time PCR reaction. Total RNA extracted from the fly brains and heads, as described above, was used to validate the expression values obtained from microarray experiments and to confirm the silencing of specific genes in the RNA-interference lines respectively. The sequence of primers are detailed in S11 Table). For each sample, 1 μg of total RNA was used for first-strand cDNA synthesis, employing 10 mM deoxynucleotides, 10 μM oligo-dT and SuperScript II (Life Technologies). qRT-PCRs were performed in triplicate in a 7500 Real-Time PCR System using SYBER Green chemistry (Promega). The 2^-ΔΔCt^ (RQ, relative quantification) method implemented in the 7500 Real Time PCR System software was used to calculate the relative expression ratio [84].

### Luciferase reporter assay

Luciferase reporter vectors containing the partial 3’-UTR of the indicated *miR-210* target genes (*soxN*, *mnd*, *Bsg*, *scrib*) were generated following PCR amplification of the 3’-UTR from Drosophila cDNA and cloned into the pmirGLO Dual-Luciferase miRNA Target Expression Vector (Promega). Where appropriate, the 3’-UTR was mutagenized at the miR-210 recognition site/s using the QuickChange Multi Site- Directed Mutagenesis kit (Stratagene-Agilent Technologies, Palo Alto, CA) following the manufacturer’s instructions. miR-210-sensor was obtained by annealing, purifying and cloning short oligonucleotides that contained three perfect miR-210 binding sites into the pmirGLO vector.0.8 x 10^6 S2R^+^ Drosophila cells were plated in 24-well plates and co-transfected with 333 ng of the pmirGLO Dual-Luciferase (Promega) construct. This contained the wild type or mutant/deleted 3’-UTRs of the indicated miR-210 potential target genes, 333 ng of the p-Act-Gal4 (a gift from Liqun Luo, Addgene plasmid #24344, [85]) and 333 ng of the pUAST-miR-210 plasmid (the same utilized to generate the flies), using Cellfectin II Reagent, following the manufacturer’s protocol (Life Technologies). Lysates were collected 24 hours after transfection and Firefly and Renilla Luciferase activities were measured with a Dual-Luciferase Reporter System (Promega). Luciferase activity was calculated by normalizing the ratio of Firefly/Renilla luciferase to negative control-transfected cells. Transfections were performed in triplicate and repeated 3 times.

### Immunohistochemistry

Flies were entrained for 3 complete days and then collected under LD or DD conditions at the indicated time points and conditions. Flies were fixed for 2 hours in PFA 4%. About 10–12 brains were dissected in PBS and then treated as previously described in [45]. The antibodies used for the immunocytochemistry experiments were anti-PDF (Developmental Studies Hybridoma Bank, dilution of 1:5,000) and anti-PER (from R. Stanewsky; 1:2,500). Alexa Fluor 488 and Alexa Fluor 568 (both from Invitrogen; 1:500) were used as secondary antibodies. The brains were observed under the ZEISS LSM 700 confocal microscope and z-series were obtained. PER intensity was quantified with ImageJ version 1.48e. The average pixel intensity for each neuron was measured together with the signal from its corresponding background area. The final amount of signal was calculated using the formula “intensity = 100×(signal − background)/background”.

### Quantification of the PDF

PDF quantification was performed on whole adult brains stained with PDF antibody. Individual images were taken of planes at different depths to create a z-series for each lobe analysed. The size of the sections forming a z-series was 0.80 ± 0.2 μm. The images were z-stacked and large LNvs were analysed using the ImageJ ITCN plugin tool for counting vesicles. The PDF signal of small-LNvs termini was quantified, as described in REF [51].

### Optomotor response

The optomotor test was performed at ZT18 following the protocol of SI setup 1 [64]. *tim-Gl4(U)*,*tub-Gl80*^*ts*^*/miR-210* flies were raised at 23°C or 29°C and then analysed at 23°C.

### Drosophila neuronal cell line

BG3-c2 Drosophila neuronal cells were obtained from DGRC and maintained in Shields and Sang M3 insect medium (Sigma) with 10% FBS (Hyclone) and 10 μg/mL insulin. Cells were co-transfected with a total of 1 μg of the following plasmids: pUAST-miR-210, p-Act-Gal4 and p-Act5-stable2-neo (a gift from Rosa Barrio & James Sutherland, Addgene plasmid # 32426, [61]) (miR-210 treated), with p-Act-Gal4 and p-Act5-stable2-neo (control) or only with pUAST-miR-Scramble, p-Act-Gal4 and p-Act5-stable2-neo (Scramble) by using Cellfectin II Reagent, following the manufacturer’s protocol (Life Technologies). GFP-positive cells were counted from 3 different fields of 3 different replicates and classified on the basis of their shape (round or neuronal). The experiment was repeated 4 times. MiR-Scramble was generated cloning 106 bp of the genomic region that contained the pre-miR-305 in a pUAST plasmid [74]. The following primers were used: *F*: *GTGTATCAACTGTCTCCCATGTCT*, *R*:*CGTATGCAAATCGCCTCATA*.

### Cytofluorimetric analysis

Transiently transfected cells were collected and stained with Annexin V Apoptosis Detection Set PE-Cyanine7 (eBioscience-ThermoFisher Scientific, Waltham, MA) and propidium iodide (Roche Biochemicals, Indianapolis, IN), again following the manufacturer’s protocol. Apoptosis cells were analyzed using Cytomics FC500 (Beckman Coulter, Brea, CA).

## Supporting information

S1 Fig *miR-210* expression levels. (A) Mature *miR-210* levels were measured by qRT-PCR at ZT0 and CT72 in *tim-Gl4(U)/+* control (dark grey) and *tim-Gl4(U)/UAS-miR-210* over-expressing fly brains (light grey). t-test was performed (*** p<0.005), and (B) at ZT6 in *yellow*^*1*^*;+;+* control (dark grey) and *yw(Ti-Gal4)miR-210*^*KO*^*;+;+* fly heads. miR-210 expression levels were normalized to 2S rRNA. RQ: Relative Quantification.
(TIF)

S2 Fig Locomotor activity of *miR-210* over-expressing flies in different clusters of neurons. Average activity of three full days displayed over the 24 hours. (A-E) *miR-210* over-expression using different Gal4 drivers. (A-C) The up-regulation of miR-210 with *cry-Gal4*, *Gal1118-Gal4* and *pdf-Gal4* significantly delayed the evening activity phase onset. (D,E) Over-expression of *miR-210* in the *miR-210* expressing tissues (*yw(Ti-Gal4)miR-210*^*KO*^*;UAS-miR-210/+*, female flies) or in the l-LNvs (*w;C929-Gal4/UAS-miR-210*) did not alter the evening activity phase. (F) *UAS-miR-210/+* control. All tested flies were males except where indicated. (ϕ: ZT evening phase activity onset ± SEM (Red line); N: number of flies analysed; y axis: activity means ± SEM; grey boxes: dark phase.).
(TIF)

S3 Fig PER expression levels of *miR-210* over-expressing flies in DD. Flies were entrained for 3 days. PER staining was performed on whole adult male brains of *tim-Gl4(U)/UAS-miR-210* over-expressing flies and controls (*tim-Gl4(U)/+*), dissected at the indicated time points. Data for each ZT were compared by t-test: *** p<0.005, ** p<0.01, * p<0.05. (s-LNvs: small ventral Lateral Neurons; l-LNvs: large ventral Lateral Neurons; 5th-LNv: 5th ventral Lateral Neurons; LNds: dorsal Lateral Neurons; DN1s: Dorsal Neurons 1; DN2s: Dorsal Neurons 2).
(TIF)

S4 Fig Effects of *miR-210* over-expressions on PDF arborisations in the Optic Lobe. Whole brains were collected at ZT0 and PDF was detected. (A-B) Confocal stack images representing l-LNvs arborisations and morphology. (A) *Gal1118-Gal4*-driven miR-210 affected both large cells body shape and PDF projections in the Optic Lobe. (B) Over-expression of *miR-210* with *pdf-Gal4* driver did not trigger an abnormal morphological phenotype of l-LNvs. Scale bar 50 μm. (OL: Optic Lobe). (C) Quantification of large LNvs PDF vesicles in *pdf-Gal4/UAS-miR-210* brains and controls (*pdf-Gal4/+*). Mean ± SD. t-test, ns: not significant.
(TIF)

S5 Fig *miR-210* over-expression altered development of l-LNvs projections. Stack of confocal images of PDF-GFP double stained brains from *UAS-GFP/UAS-miR-210;Gal1118-Gal4/+* flies. The l-LNvs GFP expression pattern was altered compared to control (*UAS-GFP/+;Gal1118-Gal4/+*) in the Optic Lobe. (Scale bar: 50 μm).
(TIF)

S6 Fig Temporal over-expression of *miR-210* in clock cells. (A) PER expression levels in *tim-Gl(U)*,*tub-Gl80*^*ts*^*/UAS-miR-210* flies and controls (*tim-Gl4)/+)*, after temporal modulation of the over-expression of *miR-210* in clock cells. Flies were raised and kept at the indicated temperature before being collected at different time points. All flies were kept under the new temperature for at least 3 complete days before they were dissected. 29°C-23°C refers to flies developed at 29°C and analysed at 23°C. 23°C-29°C refers to flies developed at 23°C and analysed at 29°C. t-test * p<0.05; ***p<0.005). (B) Sleep analysis performed in flies with large PDF aberrant arborisations (*tim-Gl4(U)*,*tub-Gl80*^*ts*^*/UAS-miR210*, 29°C-23°C) compared to controls (*tim-Gl4(U)*,*tub-Gl80*^*ts*^*/UAS-miR210*, 23°C-23°C). t-test * p<0.05; ns: not significant).
(TIF)

S7 Fig Expression levels of *echinus*, *SoxNeuro* and *minidiscs* in adult fly brains over-expressing *miR-210* in the *tim*-expressing cells (*tim-Gl4(U)/UAS-miR-210*, light grey), sampled at ZT0, with respect to their controls (*tim-Gl4(U)/+*, dark grey). Three independent experiments were performed in triplicate. The results are shown as relative expression ratios obtained with the 2^-ΔΔCt^ method ± SD. *RP49* was used as reference. (RQ: Relative quantification, t-test, *** p< 0.005; ** p< 0.01)
(TIF)

S8 Fig Validation of RNAi lines. *tim-Gl4(U)-*driven expression of the selected UAS-RNAi lines. qRT-PCR quantifications of *mnd* and *SoxN* transcripts were performed in adult fly heads collected at ZT0. Three independent experiments were performed in triplicate. The results are shown as relative expression ratios obtained with the 2-ΔΔCt method ± SD. RP49 was used as reference. (RQ: Relative quantification, t-test, *** p< 0.005; * p< 0.05).
(TIF)

S9 Fig PDF staining of large LNvs. Confocal stack images representing l-LNvs arborisations and morphology. Whole brains were collected at ZT0 and PDF was detected. The *in vivo* down-regulation of *mnd* and *SoxN*, in the *tim*-expressing neurons was not sufficient to phenocopy the star shaped l-LNvs caused by *miR-210* overexpression as shown in Fig 5. (OL: Optic Lobe; Scale bar: 50 μm).
(TIF)

S10 Fig Molecular and behavioural features of *tim-Gl4(U)/UAS-miR-210*.*5* over-expressing flies. Preliminary results showing (A) mature miR-210 levels, measured by qRT-PCR, in *tim-Gl4(U)/+*, *UAS-miR-210*.*5/+* and *w*^*1118*^ controls (dark grey) and *tim-Gl4(U)/UAS-miR-210*.*5* over-expressing fly heads (light grey), collected at ZT0. miR-210 expression levels were normalized to 2S rRNA. (B) Flies over-expressing the *UAS-miR-210*.*5* transgene in all clock cells (*tim-Gl4(U)/UAS-miR-210*.*5*) showed aberrant PDF arborisations in the optic lobes compared to controls (*UAS-miR-210*.*5/+*). Cell bodies of the large LNvs showed a star shape (arrow). (C) PER expression levels in *tim-Gl4(U)/UAS-miR-210*.*5* over-expressing flies and control (*tim-Gl4(U)/+*). Flies were entrained for 3 days. PER-PDF staining was performed on whole adult male brains dissected at ZT0 and ZT12. PER levels were higher in flies over-expressing *UAS-miR-210*.*5* (light grey) compared to controls (dark grey). No PER staining was detected at ZT12. (s-LNvs: small ventral Lateral Neurons; 5^th^-LNvs: 5^th^ ventral Lateral Neuron; l-LNvs: large ventral Lateral Neurons; LNds: dorsal Lateral Neurons; DN1s: Dorsal Neurons 1; DN2s: Dorsal Neurons 2). (D) Optomotor responses of *tim-Gl4(U)/UAS-miR-210*.5 flies compared to controls (*tim-Gl4(U)/+* and *UAS-miR-210*.*5/+*) kept at 23°C. miR-210 over-expressing flies (light grey) showed a significant reduction in the optomotor response compared to controls (dark grey). (t-test *** p<0.005; n: number of flies analysed).
(TIF)

S1 Table Morning index and evening phase onset of *miR-210* over-expressing flies. LD locomotor activity of *miR-210* over-expressing flies. Different Gal4 lines were crossed to the *UAS-miR-210* line. The progeny was monitored for three days in LD 12:12. Males were tested when the over-expression was performed with the following drivers: *cry-gal4*, *Gal1118-Gal4*, *pdf-gal4* and *C929-Gal4*, and for their respective controls. Females were monitored when *miR-210* was up-regulating with the *yw(Ti-Gal4)miR-210*^*KO*^ driver (*yw(Ti-Gal4)miR-210*^*KO*^*/w;UAS-miR-210)* and for controls (yellow^1^/*w*;+/+ and *yw (Ti-Gal4)miR-210*^*KO*^*/w;+;+* flies).^a,b^ p<0.005 vs both parental controls; ^c^ p<0.05 vs *pdf-Gal4*, p<0.005 vs *UAS-miR-210*. MI: Morning index. N: number of flies analysed. The LD E Onset (LD evening activity onset, ZT) was calculated as described in the Section Methods. Mann-Whitney U Test was performed. The experiments were performed at 23°C.
(DOCX)

S2 Table Locomotor activity of flies over-expressing *miR-210* in different neuronal clusters. *miR-210* over-expression using different Gal4 drivers. The progeny was monitored for three days in LD 12:12 and seven days in DD. Males were tested when the over-expression was performed with the following drivers: *cry-gal4*, *Gal1118-Gal4*, *pdf-gal4* and *C929-Gal4*, and for their respective controls. Females were monitored when miR-210 was up-regulating with the *yw(Ti-Gal4)miR-210*^*KO*^ driver (*yw(Ti-Gal4)miR-210*^*KO*^*/w;UAS-miR-210)* and for controls (yellow^1^/*w*;+/+ and *yw (Ti-Gal4)miR-210*^*KO*^*/w;+;+* flies). Period values (τ) were averaged over all rhythmic flies per genotype. R: rhythmic flies; MA (morning anticipation) and EA (evening anticipation) were detected, fly-by-fly, examining the bout of activity prior to light transitions as in [45]. The experiments were performed at 23°C.
(DOCX)

S3 Table Differentially expressed genes at ZT0. A list of 2,376 differentially expressed genes (941 up-regulated and 1,435 down-regulated) in miR-210 over-expressing flies (*tim-Gl4(U)/UAS-miR-210*) sampled at ZT0 and controls (*tim-Gl4(U)/+*), considering a 7.0% false discovery rate. The expression levels of each transcript were calculated as log2(miR-210/CTRL).
(XLSX)

S4 Table List of putative targets of *miR-210*. A list of 78 differentially expressed genes that are predicted to be putative targets of *miR-210* by TargetScan 6.2 and/or mirSVR algorithms. The expression levels of each transcript were calculated as log2(*miR-210*/CTRL).
(XLSX)

S5 Table Neuron development category. A list of 54 differentially expressed genes, within the “Neuron development” biological category (GO:0048666, BH adjusted p-value: 0.096) using the DAVID annotation tool. The expression levels at ZT0 of each transcript were calculated as log2(*miR-210*/CTRL).
(XLSX)

S6 Table Circadian rhythm category. A list of 22 differentially expressed genes, within the “Circadian rhythms” biological category (GO:0007623, BH adjusted p-value: 0.095) using the DAVID annotation tool. The expression levels at ZT0 and ZT12 of each transcript were calculated as log2(*miR-210*/CTRL).
(XLSX)

S7 Table Small GTPAses category. A list of 19 differentially expressed genes within the “small GTPase mediated signal transduction” biological category (GO:0007264, BH adjusted p-value: 0.098) using the DAVID annotation tool. The expression levels at ZT0 of each transcript were calculated as log2(*miR-210*/CTRL).
(XLSX)

S8 Table Photoreception category. A list of 12 differentially expressed genes grouped inside the “Photoreception” biological category, selected by a manual literature search. The expression levels at ZT0 of each transcript were calculated as log2(*miR-210*/CTRL).
(XLSX)

S9 Table Differentially expressed genes at ZT12. A list of 3,943 differentially expressed genes (1,720 up-regulated and 2,223 down-regulated) in *miR-210* overexpressing flies (*tim-Gl4(U)/UAS-miR-210*) sampled at ZT12, compared to controls (*tim-Gl4(U)/+*), considering a 7.0% false discovery rate. The expression levels of each transcript were calculated as log2(miR-210/CTRL).
(XLSX)

S10 Table Locomotor activity of flies over-expressing a second *UAS-miR-210*.*5* insertion line. (R: rhythmic flies; MA: morning anticipation; EA: evening anticipation).
(DOCX)

S11 Table List of qRT-PCR primers used in this study. (DOCX)

S12 Table Numerical data on which each graph was based, and pertinent statistical significance values. (XLSX)
