# Supplementary figures and images for: Modulation of miR-210 alters phasing of circadian locomotor activity and impairs projections of PDF clock neurons in Drosophila melanogaster
Source: PLoS Genet. 2018 Jul 16;14(7):e1007500. doi: 10.1371/journal.pgen.1007500 (PMC6062148; doi:10.1371/journal.pgen.1007500)

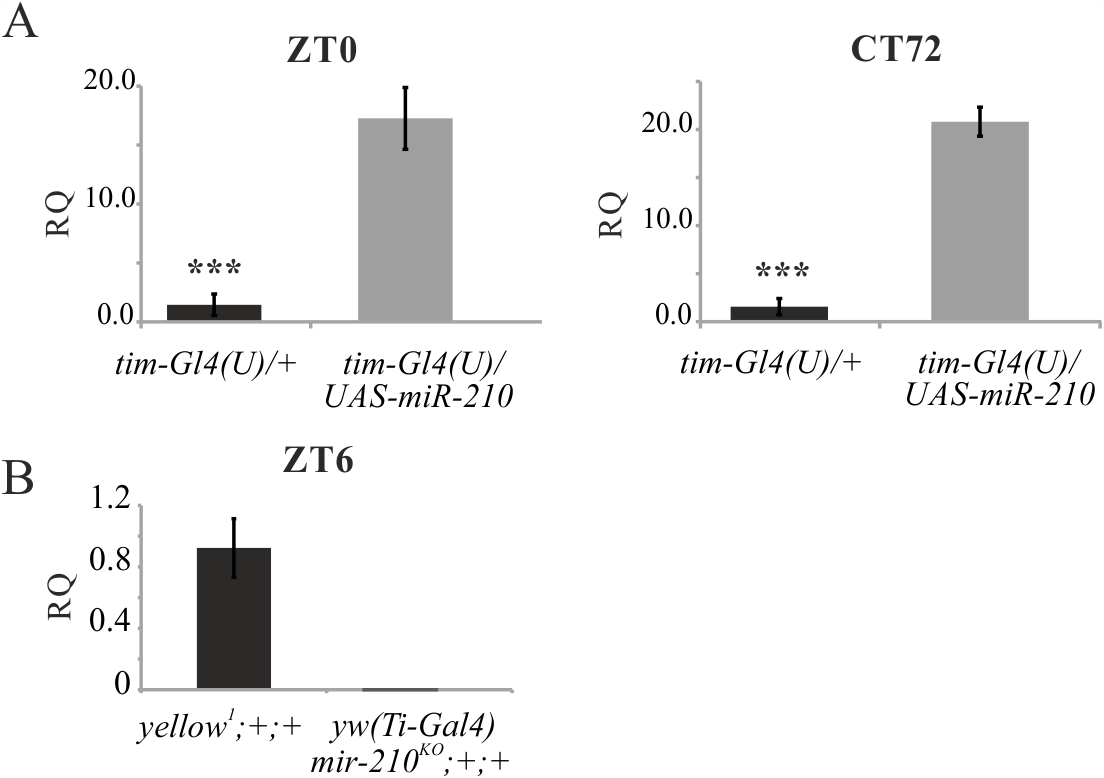

Supplement: S1 Fig — (A) Mature miR-210 levels were measured by qRT-PCR at ZT0 and CT72 in tim-Gl4(U)/+ control (dark grey) and tim-Gl4(U)/UAS-miR-210 over-expressing fly brains (light grey). t-test was performed (*** p<0.005), and (B) at ZT6 in yellow1;+;+ control (dark grey) and yw(Ti-Gal4)miR-210KO;+;+ fly heads. miR-210 expression levels were normalized to 2S rRNA. RQ: Relative Quantification. (TIF) [file pgen.1007500.s001.tif]

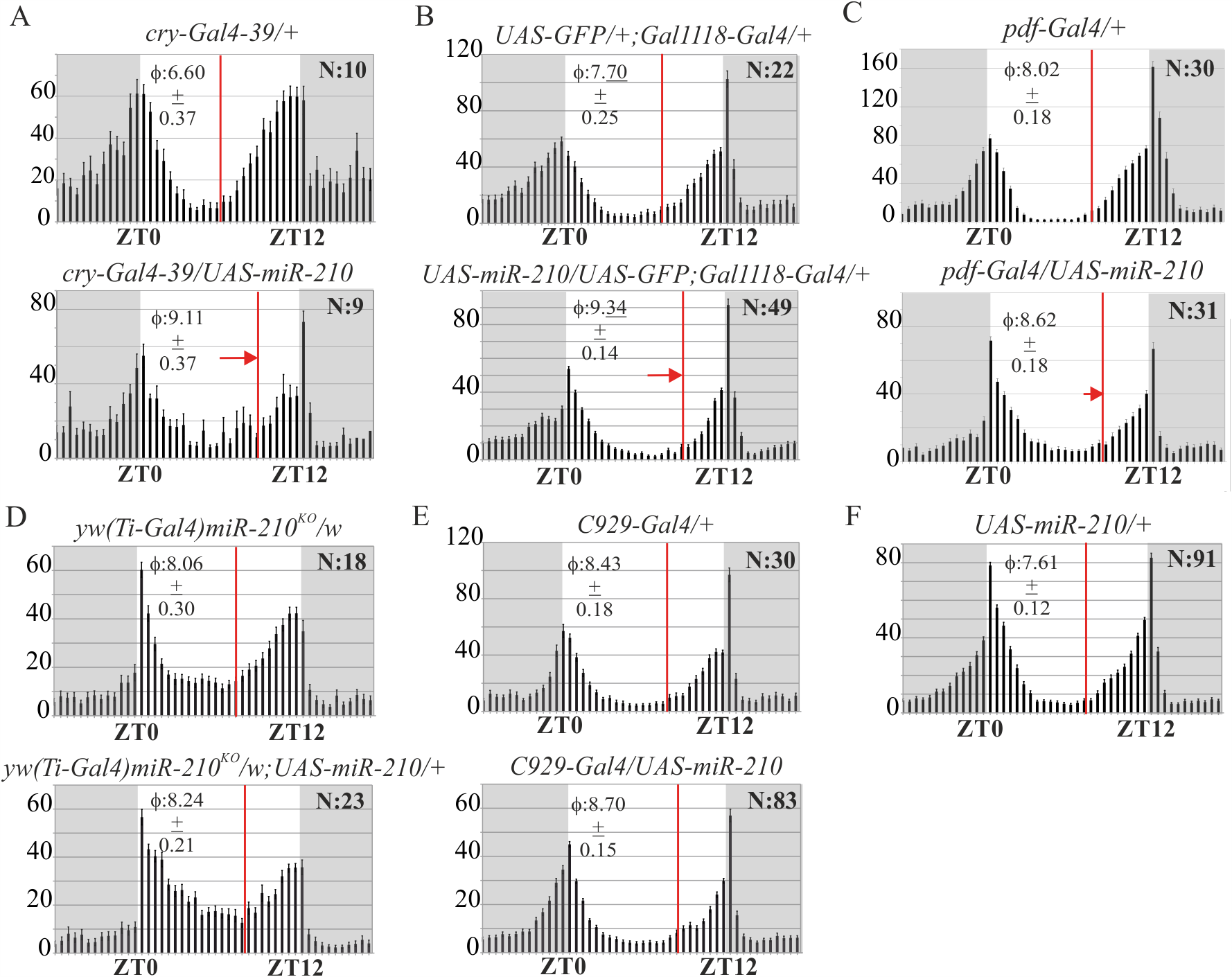

Supplement: S2 Fig — Average activity of three full days displayed over the 24 hours. (A-E) miR-210 over-expression using different Gal4 drivers. (A-C) The up-regulation of miR-210 with cry-Gal4, Gal1118-Gal4 and pdf-Gal4 significantly delayed the evening activity phase onset. (D,E) Over-expression of miR-210 in the miR-210 expressing tissues (yw(Ti-Gal4)miR-210KO;UAS-miR-210/+, female flies) or in the l-LNvs (w;C929-Gal4/UAS-miR-210) did not alter the evening activity phase. (F) UAS-miR-210/+ control. All tested flies were males except where indicated. (ϕ: ZT evening phase activity onset ± SEM (Red line); N: number of flies analysed; y axis: activity means ± SEM; grey boxes: dark phase.). (TIF) [file pgen.1007500.s002.tif]

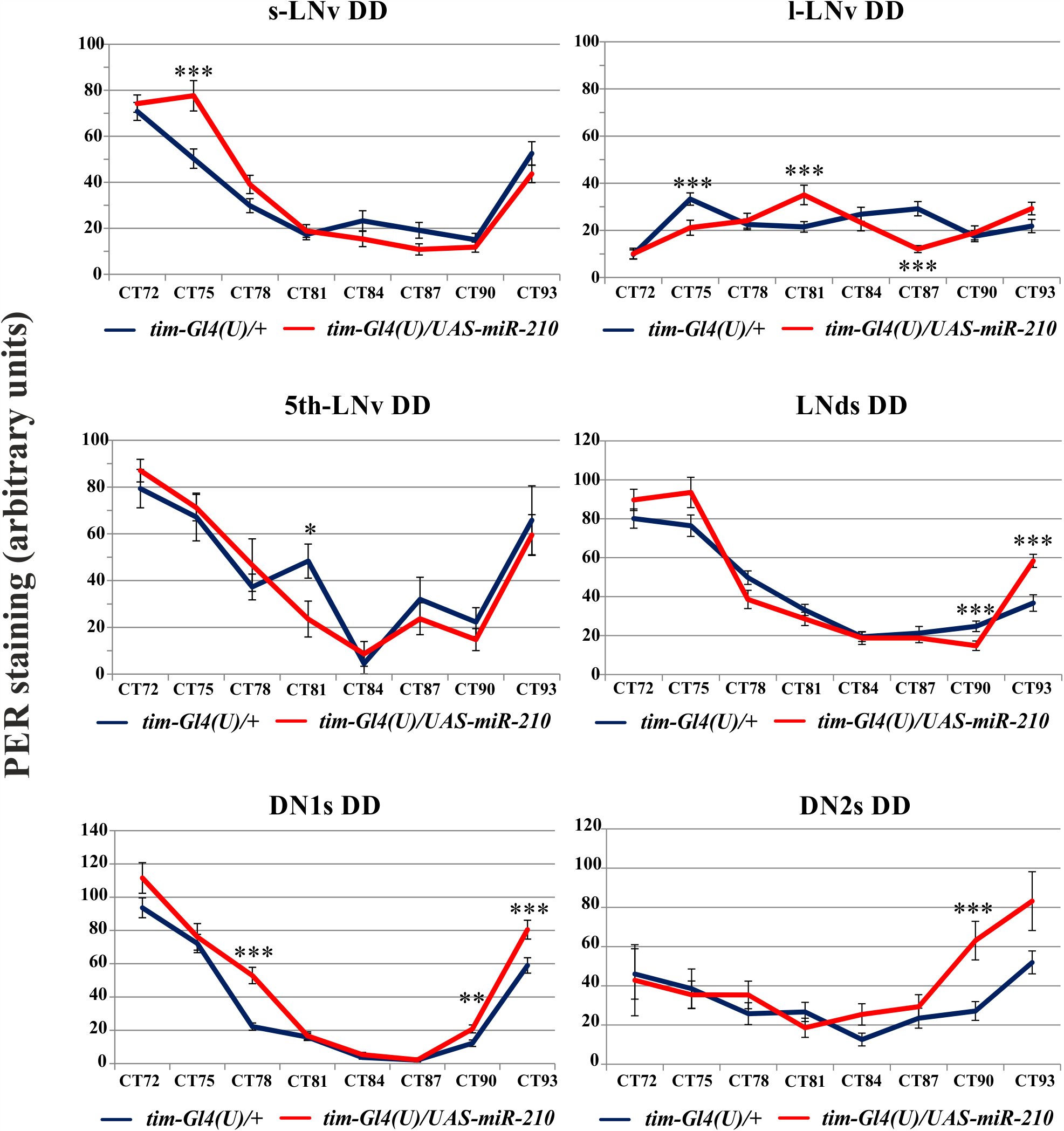

Supplement: S3 Fig — Flies were entrained for 3 days. PER staining was performed on whole adult male brains of tim-Gl4(U)/UAS-miR-210 over-expressing flies and controls (tim-Gl4(U)/+), dissected at the indicated time points. Data for each ZT were compared by t-test: *** p<0.005, ** p<0.01, * p<0.05. (s-LNvs: small ventral Lateral Neurons; l-LNvs: large ventral Lateral Neurons; 5th-LNv: 5th ventral Lateral Neurons; LNds: dorsal Lateral Neurons; DN1s: Dorsal Neurons 1; DN2s: Dorsal Neurons 2). (TIF) [file pgen.1007500.s003.tif]

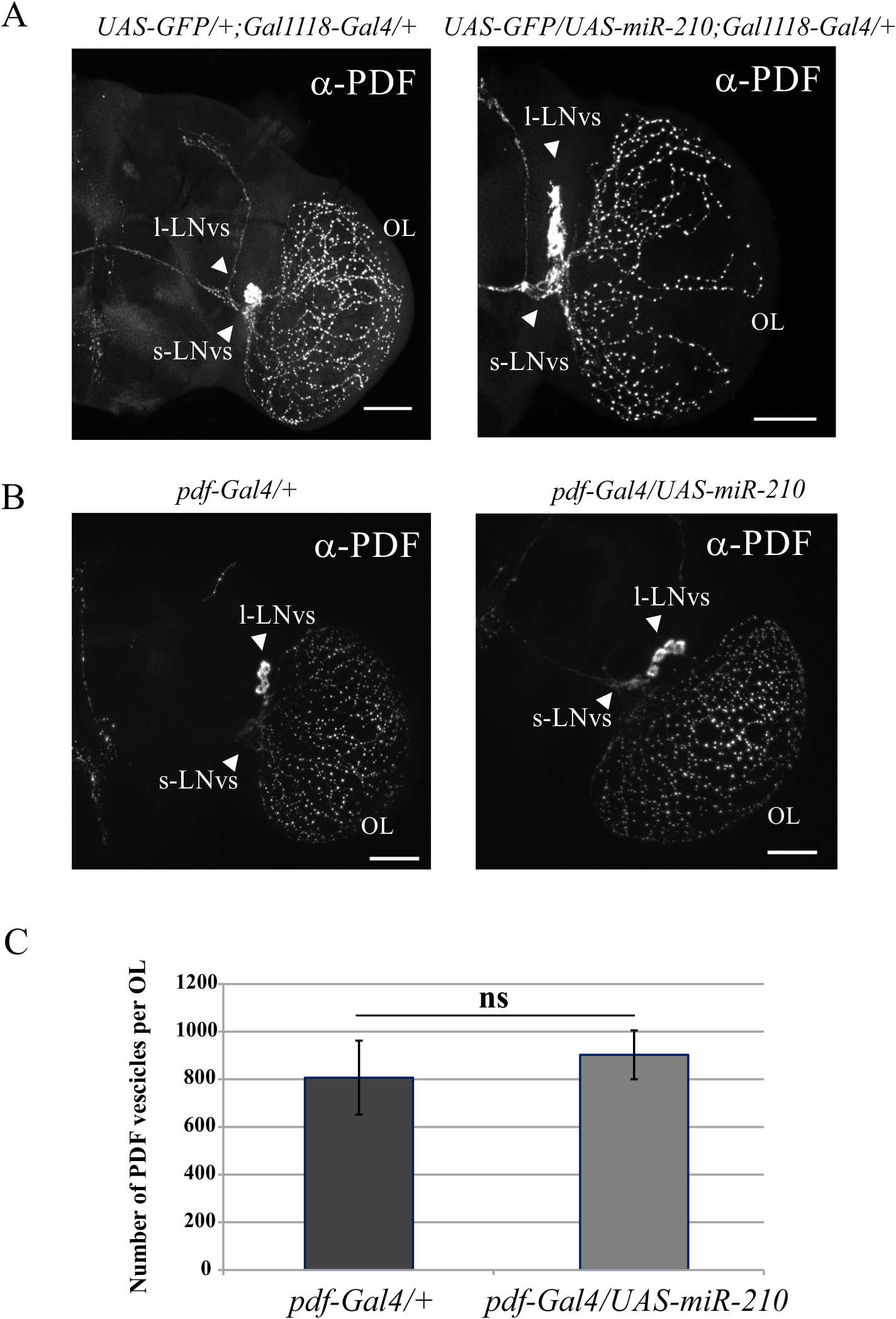

Supplement: S4 Fig — Whole brains were collected at ZT0 and PDF was detected. (A-B) Confocal stack images representing l-LNvs arborisations and morphology. (A) Gal1118-Gal4-driven miR-210 affected both large cells body shape and PDF projections in the Optic Lobe. (B) Over-expression of miR-210 with pdf-Gal4 driver did not trigger an abnormal morphological phenotype of l-LNvs. Scale bar 50 μm. (OL: Optic Lobe). (C) Quantification of large LNvs PDF vesicles in pdf-Gal4/UAS-miR-210 brains and controls (pdf-Gal4/+). Mean ± SD. t-test, ns: not significant. (TIF) [file pgen.1007500.s004.tif]

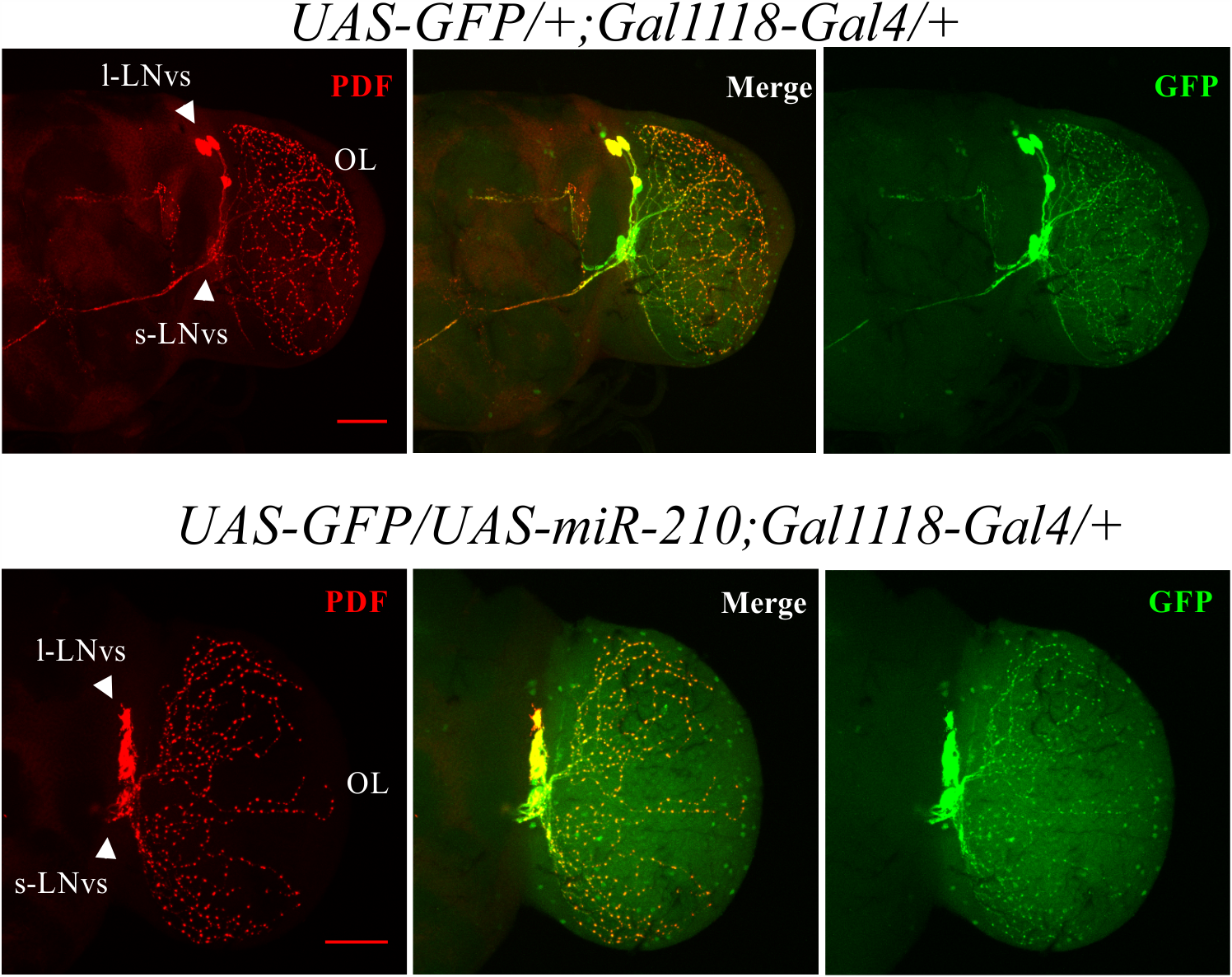

Supplement: S5 Fig — Stack of confocal images of PDF-GFP double stained brains from UAS-GFP/UAS-miR-210;Gal1118-Gal4/+ flies. The l-LNvs GFP expression pattern was altered compared to control (UAS-GFP/+;Gal1118-Gal4/+) in the Optic Lobe. (Scale bar: 50 μm). (TIF) [file pgen.1007500.s005.tif]

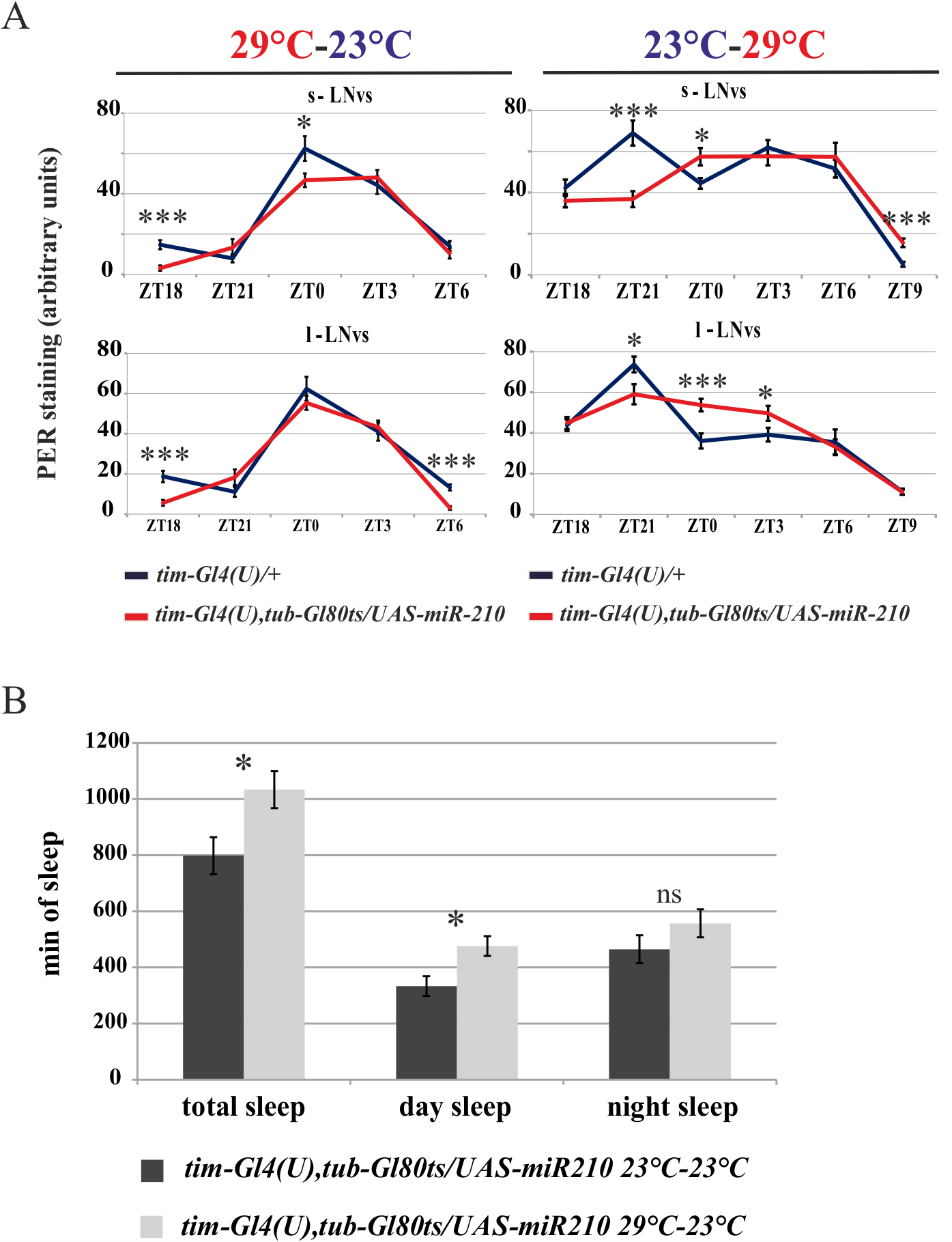

Supplement: S6 Fig — (A) PER expression levels in tim-Gl(U),tub-Gl80ts/UAS-miR-210 flies and controls (tim-Gl4)/+), after temporal modulation of the over-expression of miR-210 in clock cells. Flies were raised and kept at the indicated temperature before being collected at different time points. All flies were kept under the new temperature for at least 3 complete days before they were dissected. 29°C-23°C refers to flies developed at 29°C and analysed at 23°C. 23°C-29°C refers to flies developed at 23°C and analysed at 29°C. t-test * p<0.05; ***p<0.005). (B) Sleep analysis performed in flies with large PDF aberrant arborisations (tim-Gl4(U),tub-Gl80ts/UAS-miR210, 29°C-23°C) compared to controls (tim-Gl4(U),tub-Gl80ts/UAS-miR210, 23°C-23°C). t-test * p<0.05; ns: not significant). (TIF) [file pgen.1007500.s006.tif]

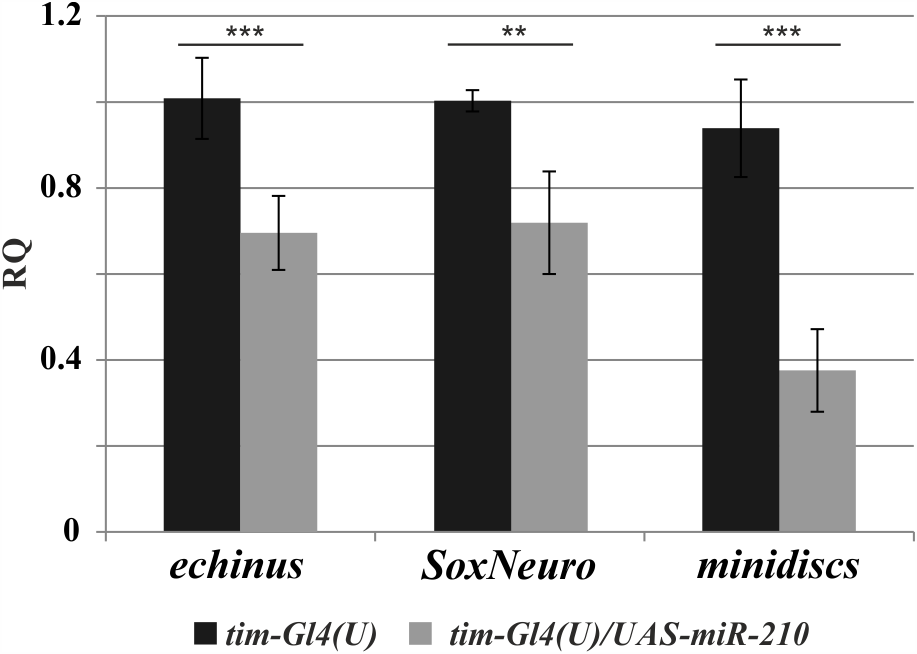

Supplement: S7 Fig — Three independent experiments were performed in triplicate. The results are shown as relative expression ratios obtained with the 2-ΔΔCt method ± SD. RP49 was used as reference. (RQ: Relative quantification, t-test, *** p< 0.005; ** p< 0.01) (TIF) [file pgen.1007500.s007.tif]

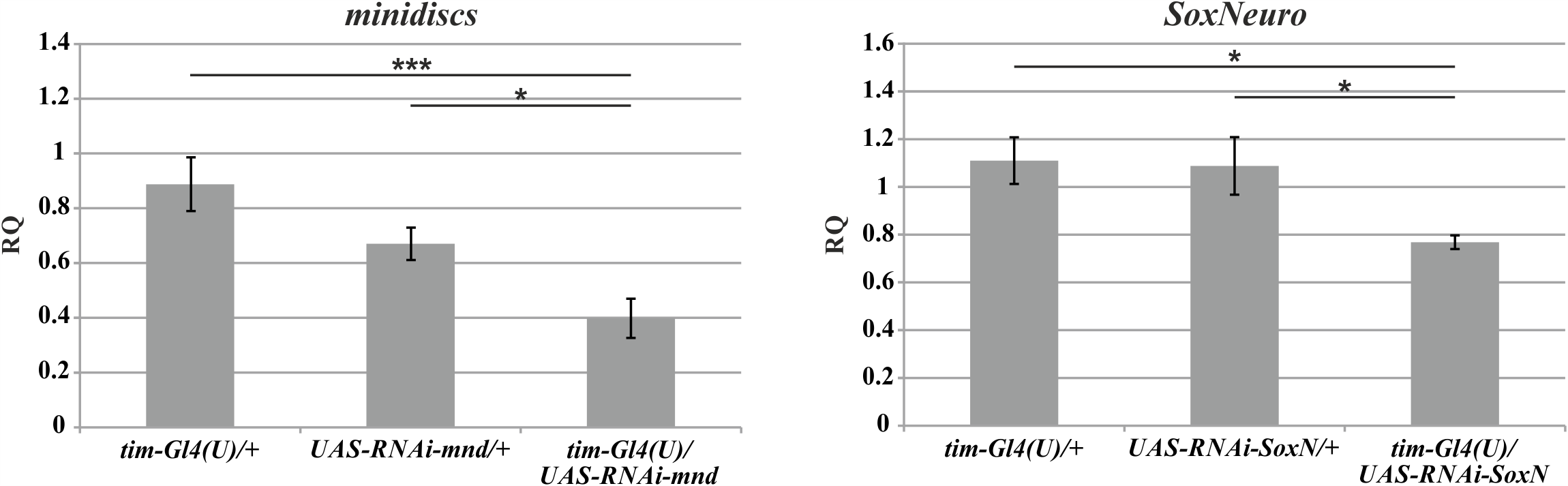

Supplement: S8 Fig — tim-Gl4(U)-driven expression of the selected UAS-RNAi lines. qRT-PCR quantifications of mnd and SoxN transcripts were performed in adult fly heads collected at ZT0. Three independent experiments were performed in triplicate. The results are shown as relative expression ratios obtained with the 2-ΔΔCt method ± SD. RP49 was used as reference. (RQ: Relative quantification, t-test, *** p< 0.005; * p< 0.05). (TIF) [file pgen.1007500.s008.tif]

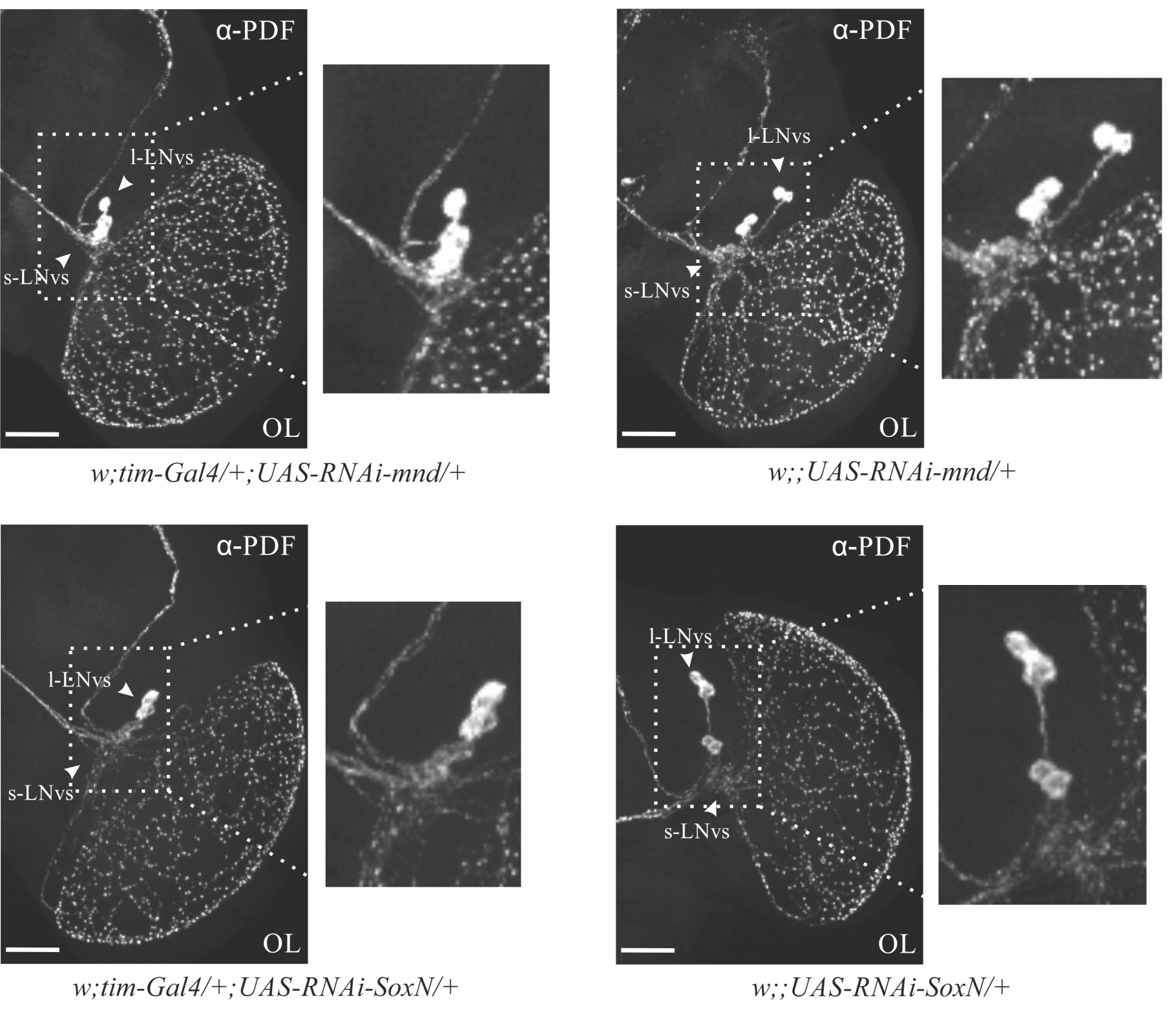

Supplement: S9 Fig — Confocal stack images representing l-LNvs arborisations and morphology. Whole brains were collected at ZT0 and PDF was detected. The in vivo down-regulation of mnd and SoxN, in the tim-expressing neurons was not sufficient to phenocopy the star shaped l-LNvs caused by miR-210 overexpression as shown in Fig 5. (OL: Optic Lobe; Scale bar: 50 μm). (TIF) [file pgen.1007500.s009.tif]

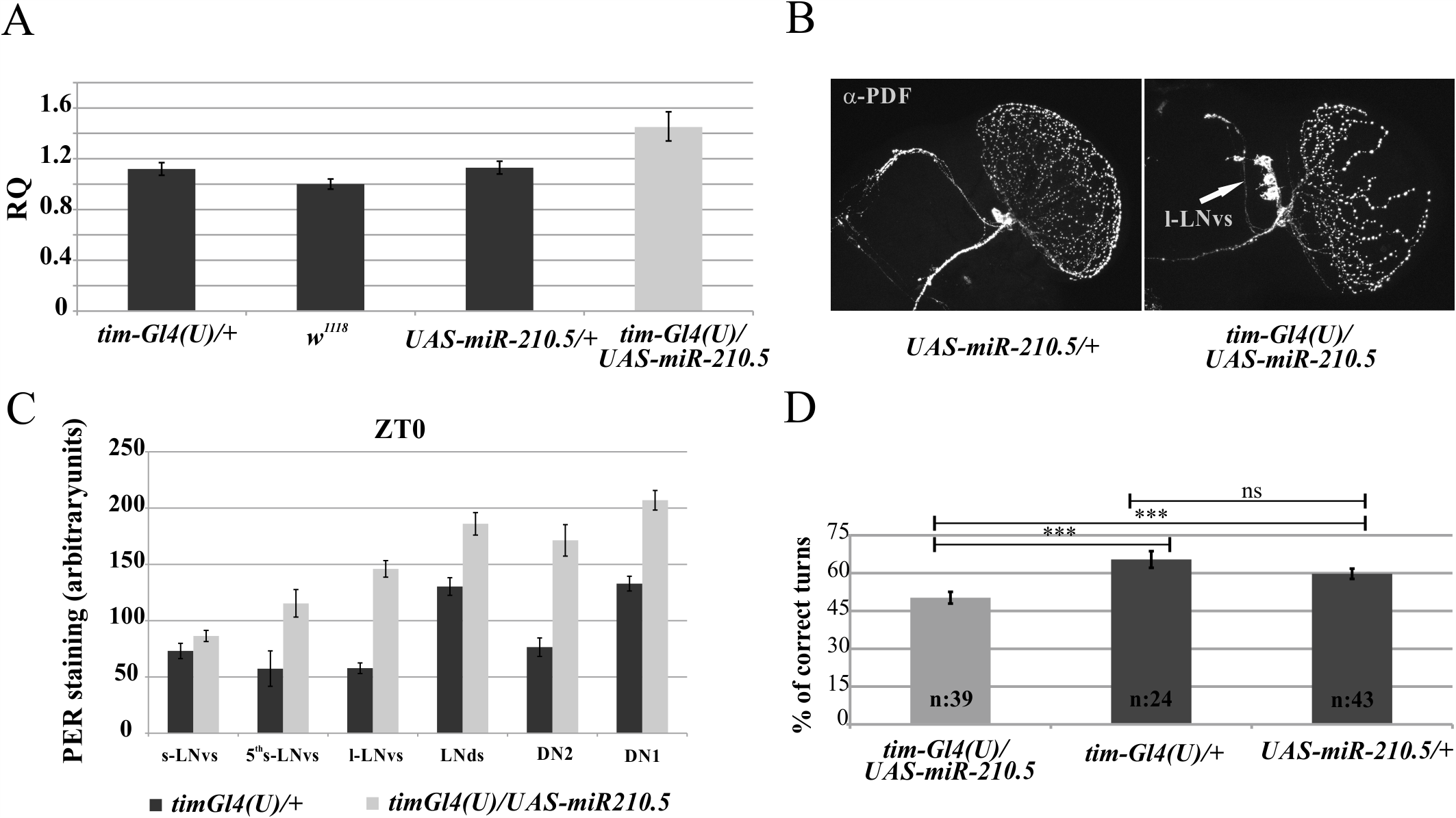

Supplement: S10 Fig — Preliminary results showing (A) mature miR-210 levels, measured by qRT-PCR, in tim-Gl4(U)/+, UAS-miR-210.5/+ and w1118 controls (dark grey) and tim-Gl4(U)/UAS-miR-210.5 over-expressing fly heads (light grey), collected at ZT0. miR-210 expression levels were normalized to 2S rRNA. (B) Flies over-expressing the UAS-miR-210.5 transgene in all clock cells (tim-Gl4(U)/UAS-miR-210.5) showed aberrant PDF arborisations in the optic lobes compared to controls (UAS-miR-210.5/+). Cell bodies of the large LNvs showed a star shape (arrow). (C) PER expression levels in tim-Gl4(U)/UAS-miR-210.5 over-expressing flies and control (tim-Gl4(U)/+). Flies were entrained for 3 days. PER-PDF staining was performed on whole adult male brains dissected at ZT0 and ZT12. PER levels were higher in flies over-expressing UAS-miR-210.5 (light grey) compared to controls (dark grey). No PER staining was detected at ZT12. (s-LNvs: small ventral Lateral Neurons; 5th-LNvs: 5th ventral Lateral Neuron; l-LNvs: large ventral Lateral Neurons; LNds: dorsal Lateral Neurons; DN1s: Dorsal Neurons 1; DN2s: Dorsal Neurons 2). (D) Optomotor responses of tim-Gl4(U)/UAS-miR-210.5 flies compared to controls (tim-Gl4(U)/+ and UAS-miR-210.5/+) kept at 23°C. miR-210 over-expressing flies (light grey) showed a significant reduction in the optomotor response compared to controls (dark grey). (t-test *** p<0.005; n: number of flies analysed). (TIF) [file pgen.1007500.s010.tif]
