# Supplementary material for: Modulation of miR-210 alters phasing of circadian locomotor activity and impairs projections of PDF clock neurons in Drosophila melanogaster
Source: PLoS Genet. 2018 Jul 16;14(7):e1007500. doi: 10.1371/journal.pgen.1007500 (PMC6062148; doi:10.1371/journal.pgen.1007500)
Supplement: S1 Table — LD locomotor activity of miR-210 over-expressing flies. Different Gal4 lines were crossed to the UAS-miR-210 line. The progeny was monitored for three days in LD 12:12. Males were tested when the over-expression was performed with the following drivers: cry-gal4, Gal1118-Gal4, pdf-gal4 and C929-Gal4, and for their respective controls. Females were monitored when miR-210 was up-regulating with the yw(Ti-Gal4)miR-210KO driver (yw(Ti-Gal4)miR-210KO/w;UAS-miR-210) and for controls (yellow1/w;+/+ and yw (Ti-Gal4)miR-210KO/w;+;+ flies).a,b p<0.005 vs both parental controls; c p<0.05 vs pdf-Gal4, p<0.005 vs UAS-miR-210. MI: Morning index. N: number of flies analysed. The LD E Onset (LD evening activity onset, ZT) was calculated as described in the Section Methods. Mann-Whitney U Test was performed. The experiments were performed at 23°C. (DOCX) [file pgen.1007500.s011.docx]

| **S1 Table. Morning Index and Evening Phase Onset of miR-210 over-expressing flies.** | | | | | | | | | | |  |  |
| --- | --- | --- | --- | --- | --- | --- | --- | --- | --- | --- | --- | --- |
|  |  |  |  |  |  |  | | |  | | |  |
| **Genotype** | **MI (n)** |  | **SEM** | **E Onset (n)** | | |  | **SEM** | |  |  |  |
|  |  |  |  |  | | |  |  | |  |  |  |
| *w;cry-Gal4/UAS-miR-210* | 0.088 (11) | ± | 0.06 | **9.11** (9)  ^a^ | | | ± | 0.37 | |  |  |  |
| *w;cry-Gal4/+* | 0.120 (72) | ± | 0.01 | 6.60 (10) | | | ± | 0.37 | |  |  |  |
| *w;UAS-miR-210/UAS-GFP;Gal1118-Gal4/+* | 0.164 (56) | ± | 0.02 | **9.34** (49) ^b^ | | | ± | 0.14 | |  |  |  |
| *w;UAS-GFP/+;Gal1118-Gal4/+* | 0.159 (23) | ± | 0.02 | 7.70 (22) | | | ± | 0.25 | |  |  |  |
| *w;pdf-Gal4/UAS-miR-210* | 0.168 (31) | ± | 0.03 | **8.62** (29) ^c^ | | | ± | 0.18 | |  |  |  |
| *w;pdf-Gal4/+* | 0.197 (30) | ± | 0.03 | 7.97 (30) | | | ± | 0.19 | |  |  |  |
| *w;C929-Gal4/UAS-miR-210* | 0.210 (90) | ± | 0.02 | 8.70 (83) | | | ± | 0.15 | |  |  |  |
| *w;C929-Gal4/+* | 0.181 (62) | ± | 0.23 | 8.43 (30) | | | ± | 0.18 | |  |  |  |
| *yw (Ti-Gal4)miR-210^KO^/w;+;+* | 0.096 (17) | ± | 0.04 | 8.06 (18) | | | ± | 0.30 | |  |  |  |
| *yellow^1^/w;+;+* | 0.139 (8) | ± | 0.04 | 9.94 (8) | | | ± | 0.30 | |  |  |  |
| *yw (Ti-Gal4)miR-210^KO^/w;UAS-miR-210/+* | 0.139 (24) | ± | 0.05 | 8.24 (23) | | | ± | 0.21 | |  |  |  |
|  |  |  |  |  | | |  |  | |  |  |  |
