# Supplementary material for: Modulation of miR-210 alters phasing of circadian locomotor activity and impairs projections of PDF clock neurons in Drosophila melanogaster
Source: PLoS Genet. 2018 Jul 16;14(7):e1007500. doi: 10.1371/journal.pgen.1007500 (PMC6062148; doi:10.1371/journal.pgen.1007500)
Supplement: S2 Table — miR-210 over-expression using different Gal4 drivers. The progeny was monitored for three days in LD 12:12 and seven days in DD. Males were tested when the over-expression was performed with the following drivers: cry-gal4, Gal1118-Gal4, pdf-gal4 and C929-Gal4, and for their respective controls. Females were monitored when miR-210 was up-regulating with the yw(Ti-Gal4)miR-210KO driver (yw(Ti-Gal4)miR-210KO/w;UAS-miR-210) and for controls (yellow1/w;+/+ and yw (Ti-Gal4)miR-210KO/w;+;+ flies). Period values (τ) were averaged over all rhythmic flies per genotype. R: rhythmic flies; MA (morning anticipation) and EA (evening anticipation) were detected, fly-by-fly, examining the bout of activity prior to light transitions as in [45]. The experiments were performed at 23°C. (DOCX) [file pgen.1007500.s012.docx]

| **S2 Table. Locomotor activity of flies over-expressing miR-210 in different neuronal clusters.** | | | | | | | | | |
| --- | --- | --- | --- | --- | --- | --- | --- | --- | --- |
| **Genotype** | **N°**  **tot** | **N°**  **alive** | **N°**  **R** | **%**  **R** | **τ** |  | **SEM** | **% MA** | **%**  **EA** |
| *w;cry-Gal4/+* | 72 | 72 | 58 | 80.56 | 24.13 | ± | 0.07 | 88.89 | 100 |
| *w;cry-Gal4/UAS-miR-210* | 11 | 11 | 6 | **54.55** | 24.83 | ± | 0.36 | 81.82 | 90.91 |
| *w;UAS-GFP/+;Gal1118-Gal4/+* | 24 | 23 | 18 | 78.26 | 24.40 | ± | 0.09 | 100 | 100 |
| *w;UAS-miR-210/UAS-GFP;Gal1118-Gal4/+* | 51 | 51 | 41 | 80.39 | 24.98 | ± | 0.06 | 86.27 | 96.08 |
| *w;pdf-Gal4/+* | 31 | 30 | 27 | 90.00 | 23.89 | ± | 0.06 | 96.67 | 100.00 |
| *w;pdf-Gal4/UAS-miR-210* | 31 | 31 | 21 | 67.74 | 24.51 | ± | 0.53 | 48.39 | 93.55 |
| *w;C929-Gal4/+* | 62 | 62 | 57 | 91.94 | 24.02 | ± | 0.04 | 95.16 | 96.77 |
| *w;C929-Gal4/UAS-miR-210* | 92 | 90 | 75 | 83.33 | 24.08 | ± | 0.06 | 80.00 | 90.00 |
| *yw (Ti-Gal4)miR-210^KO^/w;+;+* | 19 | 18 | 17 | 94.44 | 23.89 | ± | 0.13 | 66.67 | 88.89 |
| *yellow^1^/w;+;+* | 8 | 8 | 8 | 100 | 23.69 | ± | 0.08 | 87.50 | 100 |
| *yw (Ti-Gal4)miR-210^KO^/w;UAS-miR-210/+* | 29 | 24 | 22 | 91.67 | 23.76 | ± | 0.11 | 66.67 | 91.67 |
|  |  |  |  |  |  |  |  |  |  |

R: rhythmic flies; MA: morning anticipation; EA: evening anticipation. MA and EA were detected, fly-by-fly, examining the bout of activity prior to light transitions (see Methods). The experiments were performed at 23°C.
