# Supplementary material for: Modulation of miR-210 alters phasing of circadian locomotor activity and impairs projections of PDF clock neurons in Drosophila melanogaster
Source: PLoS Genet. 2018 Jul 16;14(7):e1007500. doi: 10.1371/journal.pgen.1007500 (PMC6062148; doi:10.1371/journal.pgen.1007500)
Supplement: S10 Table — (R: rhythmic flies; MA: morning anticipation; EA: evening anticipation). (DOCX) [file pgen.1007500.s020.docx]

| **S10 Table. Locomotor activity of flies over-expressing a second UAS-miR-210.5 insertion line.** | | | | | | | | | |
| --- | --- | --- | --- | --- | --- | --- | --- | --- | --- |
| **Genotype** | **N°**  **tot** | **N°**  **alive** | **N°**  **R** | **%**  **R** | **τ** |  | **SEM** | **% MA** | **%**  **EA** |
| *w;tim-Gl4(U)/UAS-miR-210.5* | 210 | 193 | 98 | **50.78** | 24.28 | ± | 0.05 | 38.34 | 87.56 |
| *w;UAS-miR-210.5/+* | 200 | 175 | 133 | 76.00 | 23.75 | ± | 0.05 | 79.43 | 93.71 |

R: rhythmic flies; MA: morning anticipation; EA: evening anticipation. MA and EA were detected, fly-by-fly, examining the bout of activity prior to light transitions. The experiments were performed at 23°C.
