# Supplementary material for: Modulation of miR-210 alters phasing of circadian locomotor activity and impairs projections of PDF clock neurons in Drosophila melanogaster
Source: PLoS Genet. 2018 Jul 16;14(7):e1007500. doi: 10.1371/journal.pgen.1007500 (PMC6062148; doi:10.1371/journal.pgen.1007500)
Supplement: S11 Table — (DOCX) [file pgen.1007500.s021.docx]

| **Gene** | **Primer** |
| --- | --- |
| *SoxNeuro* | F: TCC CTT GAT TTC CCG CTT GT |
|  | R: TGT CCC CGT AAT TGG CAA CA |
| *echinus* | F: CTG CCC TTC ACC CAG ATG |
|  | R: ACT TTT CTG GGA GGT CAG AGC |
| *minidisc* | F: GGA CAA TCC CTC ATC GTT TG |
|  | R: CCT GAT TTG GGT ATC ATC GTG |
| *rp49* | F: TCGGTTACGGATCGAACAA |
|  | R: GACAATCTCCTTGCGCTTCT |
|  |  |
|  |  |
|  |  |
|  |  |
|  |  |
|  |  |
|  |  |
|  |  |
